# Multi-omics Analysis of Liver Infiltrating Macrophages Following Ethanol Consumption

**DOI:** 10.1038/s41598-019-43240-4

**Published:** 2019-05-23

**Authors:** John O. Marentette, Meng Wang, Cole R. Michel, Roger Powell, Xing Zhang, Nichole Reisdorph, Kristofer S. Fritz, Cynthia Ju

**Affiliations:** Skaggs School of Pharmacy and Pharmaceutical Sciences, University of Colorado Anschutz Medical Campus, Aurora, CO 80045 USA

**Keywords:** Metabolomics, Proteomics, Monocytes and macrophages

## Abstract

Alcoholic liver disease (ALD) is a significant health hazard and economic burden affecting approximately 10 million people in the United States. ALD stems from the production of toxic-reactive metabolites, oxidative stress and fat accumulation in hepatocytes which ultimately results in hepatocyte death promoting hepatitis and fibrosis deposition. Monocyte-derived infiltrating Ly6C^hi^ and Ly6C^low^ macrophages are instrumental in perpetuating and resolving the hepatitis and fibrosis associated with ALD pathogenesis. In the present study we isolated liver infiltrating macrophages from mice on an ethanol diet and subjected them to metabolomic and proteomic analysis to provide a broad assessment of the cellular metabolite and protein differences between infiltrating macrophage phenotypes. We identified numerous differentially regulated metabolites and proteins between Ly6C^hi^ and Ly6C^low^ macrophages. Bioinformatic analysis for pathway enrichment of the differentially regulated metabolites showed a significant number of metabolites involved in the processes of glycerophospholipid metabolism, arachidonic acid metabolism and phospholipid biosynthesis. From analysis of the infiltrating macrophage proteome, we observed a significant enrichment in the biological processes of antigen presentation, actin polymerization and organization, phagocytosis and apoptotic regulation. The data presented herein could yield exciting new research avenues for the analysis of signaling pathways regulating macrophage polarization in ALD.

## Introduction

Alcoholic liver disease (ALD) affects approximately 10 million people in the United States and is a significant economic burden and public health hazard^1^. The pathogenesis of ALD stems from the production of toxic-reactive metabolites, reactive oxygen and nitrogen species (ROS and RNS), and oxidative stress associated with the metabolism of ethanol in hepatocytes^2^. Fat accumulation in hepatocytes (steatosis) is the earliest histopathological change in the liver associated with alcohol intake^3^. Continued steatosis results in hepatocyte death via apoptosis and necrosis which promotes inflammation and fibrosis formation^4,5^. A large number of individuals who develop fatty liver suffer no further complications while others progress from steatosis to hepatitis (liver inflammation). Persistent hepatitis and hepatocyte death can result in scar formation in the liver (cirrhosis) resulting in impaired liver function and altered architecture^6^. Persistent cirrhosis can ultimately lead to hepatocellular carcinoma and liver failure^7^.

Macrophages are instrumental in promoting and resolving the hepatitis and fibrosis associated with ALD as evidenced by clinical observations that macrophage inflammatory genes are upregulated in ALD and cirrhosis patients^8^. Furthermore, hepatic macrophage activation and enhanced production of tumor necrosis factor α (TNFα), interleukin (IL)-6, chemokine (C-C motif) ligand 2 (CCL2) and ROS is elicited with ethanol administration in ALD animals^9,10^. Kupffer cells (KC), the liver resident macrophages, account for approximately 90% of the macrophage population in the healthy liver^11^. KC are primarily involved in the maintenance of tissue homeostasis by serving as immune sentinels sensing pathogens, antigens or damaged cells through interactions with numerous cell surface receptors to initiate and potentiate the inflammatory response^12^. The immune response to liver injury is initiated through the production of pro-inflammatory cytokines, IL-1β and TNFα by KC. Additionally, KC produce chemokines, such as CCL2, which induces the recruitment of additional inflammatory cells, such as monocytes, to the site of injury^13^. Inflammation progresses with the chemotactic recruitment of Ly6C^+^ monocytes to inflamed tissue that differentiate into Ly6C^hi^ infiltrating macrophages (IMs)^13^. During acute or chronic liver injury, the macrophage subtype promoting inflammation in the liver are Ly6C^hi^ monocyte-derived macrophages^14,15^. Ly6C^hi^ macrophages exert pro-inflammatory, tissue-destructive responses as well as releasing pro-fibrotic mediators, such as IL-1β, platelet-derived growth factor (PDGF), connective tissue growth factor (CTGF) and transforming growth factor (TGF) β which activate hepatic stellate cells to deposit extracellular matrix and stimulate fibrosis formation^16–19^. While Ly6C^hi^ macrophages initially exert pro-fibrotic and pro-inflammatory function they can differentiate into Ly6C^low^ macrophages to facilitate tissue repair and inflammation resolution^20,21^.

Macrophages represent an incredibly diverse cell type which, depending on tissue micro-environmental cues, switch from a pro- to anti-inflammatory phenotype in the progression of various diseases. The remarkable heterogeneity of macrophages is exemplified by their often opposing roles in a variety of diseases. For instance, pro-inflammatory macrophages are important in the elimination of extracellular pathogens, but are instrumental in the pathogenesis of atherosclerosis, autoimmune and metabolic diseases^22^. Anti-inflammatory macrophages are instrumental in wound healing and inflammation resolution but when not properly regulated, factor into the pathogenesis of asthma, fibrosis and cancer development^23,24^. During the progression of ALD, macrophages actively promote and resolve the inflammatory response, rendering therapeutic targeting of macrophages a significant challenge. Therefore, a thorough analysis of the metabolic and protein differences between Ly6C^hi^ and Ly6C^low^ infiltrating macrophages following ethanol consumption is imperative in understanding the signaling pathways governing macrophage phenotypic switching. This mechanism could be harnessed for targeted therapeutic manipulation of macrophage populations in the liver.

In the current study, we isolated Ly6C^hi^ and Ly6C^low^ macrophages from the livers of ethanol-fed mice and subjected the isolated cells to metabolomic and proteomic analysis to achieve an integrated bioinformatics approach. Here, we present an in-depth analysis of the altered metabolome and proteome between Ly6C^hi^ and Ly6C^low^ liver infiltrating macrophages following ethanol consumption. The data herein elucidates novel signaling mechanisms governing macrophage phenotypic switching, with the potential for opening new avenues for therapeutic targeting macrophage polarization in ameliorating ALD progression.

## Results

### Comparative Metabolomic Analysis of Ly6C^hi^ and Ly6C^low^ Infiltrating Macrophages Following Ethanol Administration

**Figure 1:**
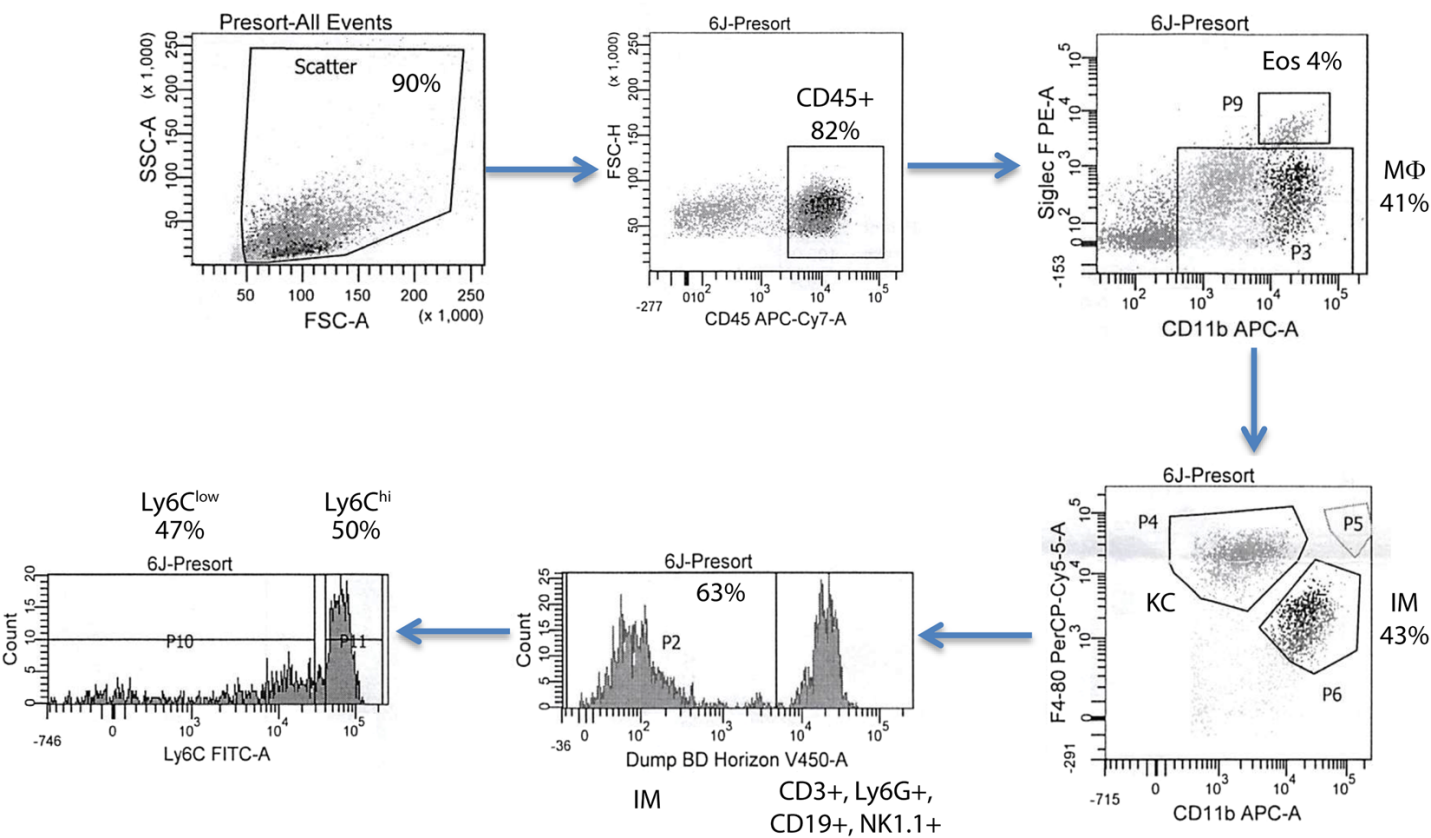
Liver macrophage flow sorting schematic. CD45 was used to select for myeloid cells. CD11b and SiglecF were used to gate out eosinophils (Eos, CD11b^+^ SiglecF^+^) from macrophages (Mϕ, CD11b^+^ SiglecF^−^). Macrophages F4/80 and CD11b were used to identify infiltrating macrophages (IM, CD11b^hi^ F4/80^Int^) from Kupffer cells (KC, CD11b^Int^ F4/80^hi^). Mixture of V450 conjugated anti-Ly6G, CD3, CD19, NK1.1 were used to gated out the neutrophils, lymophocytes and Nature Killer cells. IM were finally separated into the two infiltrating macrophage phenotypes based on expression level of Ly6C: Ly6C^hi^ and Ly6C^low^.

**Figure 2:**
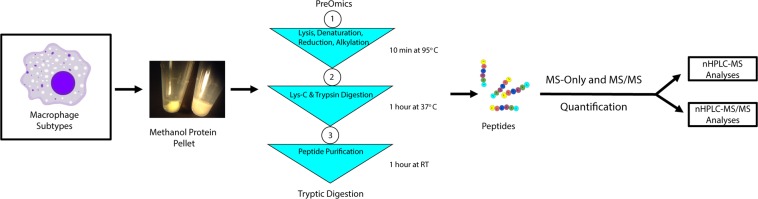
Liver macrophage metabolomics and proteomics sample preparation.

**Figure 3:**
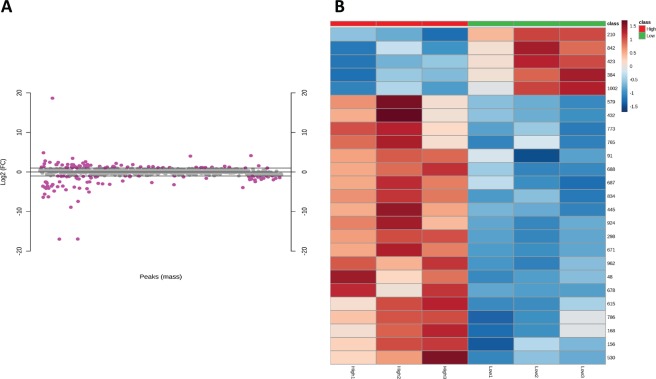
Significantly alter metabolites between Ly6C^hi^ and Ly6C^low^ macrophages. (**A**) Log2 fold change of significantly altered metabolites (n = 3 in each experiment). The pink dots represent the significant metabolites. (**B**) Heat map of significantly altered metabolites (n = 3 in each experiment). Metabolites are significant with a fold change +/− 1.5 and t-test p < 0.05 when comparing Ly6C^hi^ and Ly6C^low^.

Infiltrating Ly6C^hi^ and Ly6C^low^ liver macrophage populations from ethanol fed mice were isolated by flow sorting (Fig. 1). Following macrophage isolation, metabolites were separated from proteins using cold methanol extraction. Following methyl-tert-butyl ether (MTBE) liquid-liquid extraction, metabolites were analyzed by mass spectrometry (Fig. 2). After performing statistical analysis of the peak height intensities in Mass Profiler Professional, the ANOVA significant metabolites were uploaded to Metaboanalyst. We identified a number of metabolites with significant fold change differences between the Ly6C^hi^ and Ly6C^low^ macrophages (Fig. 3). From the metabolite analysis, we observed 102 significantly altered metabolites between the macrophage subtypes (Table 1). In the lipid positive fraction, we detected 58 differentially regulated metabolites with 39 upregulated and 19 downregulated in the Ly6C^low^ compared to the Ly6C^hi^ macrophages. From the lipid negative fraction, we measured 30 differentially regulated metabolites with 15 upregulated and 15 downregulated in the Ly6C^low^ compared to the Ly6C^hi^ macrophages. In the aqueous fraction, we detected 14 differentially regulated metabolites with 8 being upregulated and 6 downregulated in Ly6C^low^ compared to the Ly6C^hi^ macrophages. Following analysis with Metaboanalyst, we performed Metabolites Biological Role (MBROLE) analysis for pathway enrichment. From the 102 significantly altered metabolites we observed 6 pathways significantly enriched of which glycerophospholipid metabolism, arachidonic acid metabolism and phospholipid biosynthesis were further analyzed for their potential role in regulating macrophage polarization. (Table 2). Ly6C^hi^ and Ly6C^low^ macrophages are significantly enriched for glycerophospholipid metabolism, metabolic pathways, arachidonic acid metabolism, linoleic metabolism and phospholipid biosynthesis with differential regulation of the metabolites involved in each functional pathway (Supplementary Table S1).

**Table 1:** Significantly altered metabolites between Ly6C^hi^ and Ly6C^low^ macrophages.

Compound	p(Low vs High)	Regulation	Fold Change	Mass	Retention Time	Metabolite ID
**Lipid Positive Metabolites**						
17-Hydroxyprogesterone 1.1479999	1.92E-09	Up	52550.8	330.2258	1.1480	C01176
MG(0:0/18:1/0:0)	4.13E-07	Up	13307.73	378.2748	2.5390	C01885
Prosafrinine	0.01746852	Up	5.11865	305.234	0.7990	LMSP01080051
Cyclopassifloside V	0.006721366	Up	4.075243	882.4456	1.3970	HMDB35947
Okadaic acid	0.005180489	Up	2.907953	848.4317	1.4090	C01945
5,8,11-Eicosatrienoic acid	0.020084225	Up	2.815632	306.2565	1.7910	HMDB10378
5,8,11-Eicosatrienoic acid Esi + 1.7910002	0.03348608	Up	2.535191	306.2554	1.7910	HMDB10378
Spectinomycin adenylate	0.017278904	Up	2.418216	683.1959	6.5740	C03580
CL(20:4/20:4/18:1/18:1)	0.022371477	Up	2.385773	1501.0281	5.2100	C05980
9R-(2-cyclopentenyl)-1-nonanol 1.4750003	0.01784647	Up	2.252583	232.1784	1.4750	LMFA05000040
CL(16:0/18:1/18:1/18:0)	0.029356718	Up	2.216448	1433.0435	5.2100	C05980
PA(18:3/18:3)	0.044420037	Up	2.105103	692.4487	3.3660	LMGP10010015
Resiniferatoxin	0.001578898	Up	2.102412	628.2697	1.4160	C09179
Narasin	0.030150319	Up	2.018768	786.5013	1.0990	HMDB30448
(R)-1-O-[b-D-Glucopyranosyl-(1–6)-b-D-glucopyranoside]-1,3-octanediol	0.008763756	Up	1.982221	470.2431	2.0950	HMDB32799
PI(16:1/0:0)	0.0099774	Up	1.978465	570.2801	1.3970	LMGP06050009
Mephentermine	0.04304003	Up	1.732651	163.1362	0.4740	C07889
2-Hexyl-4,5-dimethyloxazole	0.039262477	Up	1.682691	181.1476	0.9940	HMDB37895
11H-14,15-EETA	0.007826052	Up	1.674163	358.2093	1.2530	C14813
3-O-Benzyl-4,5-O-(1-methylethyldiene)-b-D-fructopyranose	0.022699697	Up	1.646057	310.1397	1.0070	
Lupinine	0.015280782	Up	1.632106	169.1493	0.9770	C10773
Perilloside C	0.03297016	Up	1.615675	338.1709	1.1480	HMDB40563
14,15-Epoxy-5,8,11-eicosatrienoic acid Esi + 1.1569998	0.03620125	Up	1.609097	320.2328	1.1570	C14771
Vitamin A	0.03911776	Up	1.604065	286.2295	2.2710	C00473
8,9,10,11-Tetrafluoro-8E,10E-dodecadien-1-ol	0.02570673	Up	1.599283	254.1293	0.9460	LMFA05000168
2,2,11,13,15,16-hexachloro-docosane-1,14-disulfate	0.03122542	Up	1.59914	728.0154	5.2090	LMFA00000019
PG(14:0/16:0)	0.047110956	Up	1.598646	716.4513	3.0500	LMGP04010022
13-L-Hydroperoxylinoleic acid	0.038854554	Up	1.579102	312.2277	1.8080	C04717
9R-(2-cyclopentenyl)-1-nonanol 6.900001	0.03706475	Up	1.574036	232.1829	6.9000	LMFA05000040
Decarbamoylneosaxitoxin	0.039842825	Up	1.572547	272.1243	0.4870	HMDB33663
Rubrobrassicin	0.019814456	Up	1.565009	757.2147	7.0090	LMPK12010026
Isovitexin 2″-O-(6‴-(E)-p-coumaroyl)glucoside	0.021801876	Up	1.5623	762.1733	7.0090	LMPK12110271
Linalyl oxide	0.034410253	Up	1.521542	170.1307	1.7150	HMDB35907
1,8-Diazacyclotetradecane-2,9-dione	0.039644323	Up	1.518688	226.1685	0.4730	C04277
3,4-Dihydrocadalene	0.010371112	Up	1.512875	200.1528	0.4720	HMDB36453
Camptothecin Esi + 1.455	0.036810648	Up	1.457777	370.0917	1.4550	C01897
Imiquimod	0.04839949	Up	1.408694	240.1345	1.1220	HMDB14862
Cycluron	0.039257277	Up	1.328519	220.1547	0.9920	C19109
7″-O-Phosphohygromycin	0.036477257	Up	1.277203	629.1862	2.9640	C03368
Dodecanol	0.04339384	Down	−1.38546	208.1831	1.1930	C02277
Aristolochic Acid	0.042970523	Down	−1.49828	341.0521	1.1540	C08469
Ceramide (d18:1/22:0) 7.501	0.01739148	Down	−1.61369	621.6092	7.5010	C00195
Cer(d18:1/24:1)	0.013293305	Down	−1.69156	647.6224	7.4810	C00195
Cer(d18:0/24:1)	0.026877573	Down	−1.70931	649.638	7.8760	C00195
2-Hydroxydecanedioic acid	0.003152832	Down	−1.9211	240.0977	0.6050	HMDB00424
PE(20:1/20:3)	0.035945572	Down	−1.95305	795.5796	6.4140	C00350
N,N,O-Tridesmethyl-tramadol	0.004404348	Down	−1.97113	221.1398	0.8020	HMDB60850
Cer(d18:1/23:0)	0.004459221	Down	−2.04499	635.6211	7.6940	C00195
Ceramide (d18:1/20:0) 7.063001	0.021412965	Down	−2.04623	593.5757	7.0630	C00195
Alpha-CEHC Esi + 0.9440002	0.01759312	Down	−2.2515	278.1496	0.9440	HMDB01518
Coenzyme Q9	0.03474693	Down	−2.26301	794.6223	8.0660	C01967
Propofol glucuronide	0.028436085	Down	−2.39699	354.1736	1.2040	HMDB60933
Colnelenic acid	0.00849326	Down	−2.50289	292.2021	1.2090	LMFA10000002
3E,7Z-Tetradecadienyl acetate	0.02242055	Down	−2.81641	252.2092	1.2060	LMFA05000348
4-methyl-tridecanedioic acid	0.017504424	Down	−2.98563	258.1843	1.0030	LMFA01170017
MG(0:0/18:4/0:0) Esi + 1.455	0.010899141	Down	−3.00273	350.2418	1.4550	C01885
MG(0:0/18:4/0:0)	0.015869742	Down	−3.90034	350.2434	1.3730	C01885
24R-methylcholest-22E-en-3β,4β,5α,6α,8β,14α,15α,25 R,26-nonol	1.38E-08	Down	−25218.3	550.3125	1.3420	LMST01031080
**Lipid Negative Metabolites**						
Compound	p(Low vs High)	Regulation	Fold Change	Mass	Retention Time	Metabolite ID
Seneciphylline	1.58E-07	Up	13797.39	333.156	0.915	C10391
PC(20:3/P-18:1) 7.3700004	9.04E-09	Up	9632.273	793.5885	7.3700004	C00157
PS(22:2/20:4)	0.00619864	Up	2.130906	863.5636	6.431	C02737
PE(20:1/20:3)	0.008398175	Up	2.125419	795.5765	6.4339986	C00350
PA(14:0/13:0)	0.018941188	Up	2.03645	614.3692	5.2680006	C00416
PE(20:2/P-18:1)	0.004769958	Up	1.966516	753.5574	7.0680003	C00350
PC(20:3/P-18:0)	0.010843969	Up	1.91783	795.6032	7.6989994	C00157
PE(14:0/22:1)	0.02230334	Up	1.846544	745.5694	5.279	C00350
PS(18:0/20:3)	0.010184665	Up	1.82344	813.5564	5.286	C02737
Ceramide (d18:1/22:0)	0.024829699	Up	1.654491	667.6106	7.5	C00195
Cer(d18:1/24:1)	0.03071489	Up	1.651767	693.6258	7.4820004	C00195
PE(24:0/P-16:0)	0.020011874	Up	1.637358	805.6088	7.485	C00350
PE(O-20:0/22:4)	0.04190944	Up	1.518955	809.6189	7.883001	C13894
PE(22:2/P-18:1)	0.026633823	Up	1.515987	781.5885	7.5039997	C00350
1-(8-[3]-ladderane-octanoyl-2-(8-[3]-ladderane-octanyl)-sn-glycerol	0.039579846	Up	1.211129	650.5179	6.34	LMGL02070009
Ubiquinone-4	0.018471733	Down	−1.27887	490.2843	2.4319997	C00399
PC(14:1/P-18:0)	0.041162275	Down	−1.38207	751.5357	5.2099996	C00157
Phytosulfokine b	0.04650307	Down	−1.43648	754.1618	1.097	HMDB29810
Rimocidine	0.03588501	Down	−1.43716	767.4112	3.0529997	C15821
Acetyl-N-formyl-5-methoxykynurenamine	0.033569902	Down	−1.47063	300.0885	1.156	C05642
alpha-Ribazole	0.04385764	Down	−1.4749	314.104	1.2270001	C05775
Ceriporic acid A	0.028160162	Down	−1.50262	326.2453	1.656	LMFA01170126
PE(14:0/16:0)	0.032299943	Down	−1.52035	663.4833	5.243	C00350
CL(18:0/18:1/18:1/18:0)	0.037032653	Down	−1.59666	1461.0708	6.4690013	C05980
CL(20:1/18:2/18:1/18:1)	0.024215354	Down	−1.66882	1525.0492	5.209	C05980
PC(14:1/P-18:0) 5.355	0.044275247	Down	−1.72501	751.5351	5.355	C00157
LysoPE(0:0/22:5)	0.049099866	Down	−1.78763	509.2879	1.6539999	C05973
PE(14:1/20:4)	0.009279267	Down	−1.84186	709.4657	1.068	C00350
CL(18:0/18:0/18:2/18:0)	0.03732978	Down	−2.06192	1457.063	5.2099996	C05980
Camptothecin	0.01822235	Down	−2.09969	348.1068	0.9259999	C01897
**Aqueous Positive**						
PC(14:0/20:1)	1.17E-06	Up	14552.3	759.577	2.7959998	C00157
LysoPE(0:0/20:4)	4.39E-08	Up	8191.464	501.2852	1.5950001	C05973
PE(18:2/18:2)	8.68E-08	Up	6742.008	739.5146	2.8740003	C00350
Ceramide(d18:1/17:0)	0.033310328	Up	3.80966	551.5272	0.8509999	C00195
Ceramide(d18:1/17:0) 0.84700006	0.030002557	Up	3.701004	533.5165	0.8470001	C00195
CE(15:0)	0.033051185	Up	2.817563	609.5802	0.856	C02530
Hydroxybutyrylcarnitine	0.049097747	Up	2.040182	247.1433	5.395	HMDB13127
L-Carnitine	0.037892483	Up	1.340674	161.1052	5.8930006	C00318
Hydrocortisone caproate	0.04131846	Down	−1.2559	442.272	0.7210001	C13422
1,4′-Bipiperidine-1′-carboxylic acid	0.00321868	Down	−1.31586	211.169	1.3240001	C16836
Methylconiine	0.022564428	Down	−1.32673	141.1508	1.441	C10159
Acetaminophen glucuronide 3.3980002	0.04299609	Down	−1.41856	348.1522	3.3980002	HMDB10316
4-Guanidinobutanoic acid	0.006786303	Down	−1.45549	145.085	4.139	C01035
5beta-Gonane	0.008270364	Down	−2.18221	254.1995	2.3449998	C19640

(n = 3 in each experiment). Metabolites were considered significant with a fold change +/− 1.5 and ANOVA p < 0.05 when comparing Ly6C^hi^ and Ly6C^low^.

**Table 2:** MBROLE functional pathway enrichment of significantly altered metabolites between Ly6C^hi^ and Ly6C^low^ macrophages.

MBROLE Pathway Enrichment Analysis						
KEGG Pathway	Glycerophospholipid metabolism	p = 0.00000015	Regulation	FC	Mass	Retention Time
Metabolite ID	Compound	p ([LOW] vs [HI])				
HMDB07879	PC(14:0/20:1)	0.00000117	Up	14552.3	759.577	2.7959998
C05973	LysoPE(0:0/20:4)	0.00000004	Up	8191.464	501.2852	1.5950001
C05980	CL(20:4/20:4/18:1/18:1)	0.02237148	Up	2.385773	1501.028	5.2099996
C05980	CL(16:0/18:1/18:1/18:0)	0.02935672	Up	2.216448	1433.044	5.2099996
C02737	PS(22:2/20:4)	0.00619864	Up	2.130906	863.5636	6.431
C00416	PA(14:0/13:0)	0.01894119	Up	2.03645	614.3692	5.2680006
C02737	PS(18:0/20:3(8Z,11Z,14Z))	0.01018467	Up	1.82344	813.5564	5.286
C05980	CL(18:0/18:1/18:1/18:0)	0.03703265	Down	−1.59666	1461.071	6.4690013
C05980	CL(20:1/18:2/18:1/18:1)	0.02421535	Down	−1.66882	1525.049	5.209
C05973	LysoPE(0:0/22:5)	0.04909987	Down	−1.78763	509.2879	1.6539999
C05980	CL(18:0/18:0/18:2/18:0)	0.03732978	Down	−2.06192	1457.063	5.2099996
HMDB09093	PE(18:2/18:2)	0.00000009	Down	−6742.01	739.5146	2.8740003
**HMDB Pathway**	**Arachidonic Acid Metabolism**	**p = 0.025**	**Regulation**	**FC**	**Mass**	**Retention Time**
**Metabolite ID**	**Compound**	**p ([LOW] vs [HI])**				
C00157	PC(14:0/20:1)	1.17E-06	Up	14552.3	759.577	2.7959998
C00157	PC(20:3/P-18:1) 7.3700004	9.04E-09	Up	9632.273	793.5885	7.3700004
C00157	PC(20:3/P-18:0)	0.010843969	Up	1.91783	795.6032	7.6989994
HMDB04693	11H-14,15-EETA	0.007826052	Up	1.674163	358.2093	1.2530001
HMDB04264	14,15-Epoxy-5,8,11-eicosatrienoic acid	0.03620125	Up	1.609097	320.2328	1.1569998
C00157	PC(14:1/P-18:0)	0.041162275	Down	−1.38207	751.5357	5.2099996
C00157	PC(14:1/P-18:0) 5.355	0.044275247	Down	−1.72501	751.5351	5.355
**HMDB Pathway**	**Phospholipid Biosynthesis**	**p = 0.0000332**	**Regulation**	**FC**	**Mass**	**Retention Time**
**Metabolite ID**	**Compound**	**p ([LOW] vs [HI])**				
C00157	PC(14:0/20:1)	1.17E-06	Up	14552.3	759.577	2.7959998
C00157	PC(20:3/P-18:1) 7.3700004	9.04E-09	Up	9632.273	793.5885	7.3700004
C00350	PE(18:2/18:2)	8.68E-08	Up	6742.008	739.5146	2.8740003
C02737	PS(22:2/20:4)	0.00619864	Up	2.130906	863.5636	6.431
C00350	PE(20:1/20:3)	0.008398175	Up	2.125419	795.5765	6.4339986
C00350	PE(20:1/20:3)	0.008398175	Up	2.125419	795.5765	6.4339986
C00416	PA(14:0/13:0)	0.01894119	Up	2.03645	614.3692	5.2680006
C00350	PE(20:2/P-18:1)	0.004769958	Up	1.966516	753.5574	7.0680003
C00157	PC(20:3/P-18:0)	0.010843969	Up	1.91783	795.6032	7.6989994
C00350	PE(14:0/22:1)	0.02230334	Up	1.846544	745.5694	5.279
C02737	PS(18:0/20:3)	0.01018467	Up	1.82344	813.5564	5.286
C00350	PE(24:0/P-16:0)	0.020011874	Up	1.637358	805.6088	7.485
C00350	PE(22:2/P-18:1)	0.026633823	Up	1.515987	781.5885	7.5039997
C00157	PC(14:1/P-18:0)	0.041162275	Down	−1.38207	751.5357	5.2099996
C00350	PE(14:0/16:0)	0.032299943	Down	−1.52035	663.4833	5.243
C00157	PC(14:1/P-18:0) 5.355	0.044275247	Down	−1.72501	751.5351	5.355
C00350	PE(14:1/20:4)	0.009279267	Down	−1.84186	709.4657	1.068

(n = 3 in each experiment). Pathway enrichment was considered significantly with a MBROLE calculated p < 0.05.

### Comparative Proteomic Analysis of Ly6C^hi^ and Ly6C^low^ Infiltrating Macrophages Following Ethanol Administration

**Figure 4:**
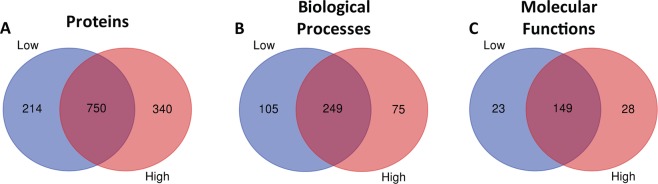
Venn Diagrams of unique protein, biological processes and molecular functions between Ly6C^hi^ and Ly6C^low^ macrophages. (**A**) Number of common and unique proteins between Ly6C^hi^ and Ly6C^low^ macrophages. (**B**) Number of common and unique biological processes between Ly6C^hi^ and Ly6C^low^ macrophages. (**C**) Number of common and unique molecular functions between Ly6C^hi^ and Ly6C^low^ macrophages. Lists of common and unique protein, biological processes and molecular functions can be found in Supplementary Tables S3–S5.

Following methanol extraction of metabolites, the remaining protein pellet was subjected to protein extraction and tryptic digested for mass spectrometry proteomics analysis. Peptides detected by mass spectrometry were searched in Spectrum Mill to determine the protein identification. We detected 1,304 proteins in Ly6C^hi^ and Ly6C^low^ macrophages with 340 and 214 proteins, respectively, uniquely expressed between macrophage subtypes (Fig. 4A). The 1,304 protein found in the Ly6C^hi^ and Ly6C^low^ macrophages were subjected to DAVID analysis. From the 1,304 proteins analyzed, we observed 429 biological processes of which 105 were unique for Ly6C^low^ and 75 for Ly6C^high^ macrophages (Fig. 4B). Furthermore, we detected 200 molecular functions from the 1,304 proteins of which 23 are unique for Ly6C^low^ and 28 for Ly6C^high^ macrophages (Fig. 4C). The UniProt accession numbers for the common and unique proteins, biological processes and molecular functions are listed in the Supplementary Information Section (Supplementary Tables S3–S5). Protein quantitative analysis of significantly altered proteins was obtained from Mass Profiler Professional and we detected 47 differentially regulated proteins between the Ly6C^hi^ and Ly6C^low^ macrophages (Table 3). The significantly altered proteins between the Ly6C^hi^ and Ly6C^low^ macrophages were analyzed using the DAVID bioinformatics resource and we observed a total of 21 biological processes and 9 molecular functions from DAVID analysis of the protein quantification obtained (Supplementary Table S2). Of the significantly enriched biological processes and molecular functions, immune processes, actin polymerization and organization, phagocytosis, apoptotic processes and antigen presentation were selected for additional literature based analysis in their potential role for regulating macrophage polarization (Table 4).

**Table 3:** Quantitative analysis of MS-only spectra of significantly altered proteins between Ly6C^hi^ and Ly6C^low^ macrophages.

Quantitative Proteomics Analysis					
Protein Name	Protein ID	Peptide #	p-value	Fold Change (Low vs High)	Regulation
Phospholipase D3	O35405	2	8.24E-09	32371.51	Up
Cathepsin L1	P06797	4	2.77E-08	5875.09	Up
Ras-related protein Rap-1b	Q99JI6	2	4.55E-02	1512.80	Up
Protein S100-A9	P31725	8	4.06E-05	32.22	Up
Protein S100-A8	P27005	5	2.17E-04	31.14	Up
Cathelin-related antimicrobial peptide	P51437	2	1.72E-05	28.13	Up
H-2 class II histocompatibility antigen, A-B alpha chain	P14434	4	4.57E-04	17.26	Up
H-2 class II histocompatibility antigen, A beta chain	P14483	4	1.85E-04	15.85	Up
Lactotransferrin	P08071	14	4.63E-04	14.67	Up
Neutrophil gelatinase-associated lipocalin	P11672	2	2.42E-03	14.47	Up
Macrophage asialoglycoprotein-binding protein 1	P49300	4	3.92E-04	6.48	Up
H-2 class II histocompatibility antigen gamma chain	P04441	4	7.13E-04	4.74	Up
CD177 antigen	Q8R2S8	3	8.01E-04	4.70	Up
Gelsolin	P13020	11	1.36E-03	3.17	Up
Transcription factor A, mitochondrial	P40630	2	9.01E-03	3.09	Up
Vasodilator-stimulated phosphoprotein	P70460	2	1.67E-02	2.91	Up
EF-hand domain-containing protein D2	Q9D8Y0	3	2.42E-02	2.79	Up
Putative phospholipase B-like 1	Q8VCI0	6	9.23E-03	2.54	Up
Chitinase-3-like protein 3	O35744	13	6.29E-03	2.54	Up
Synaptosomal-associated protein 23	O09044	2	4.40E-03	2.51	Up
Low affinity immunoglobulin gamma Fc region receptor II	P08101	4	1.73E-02	2.46	Up
Histone H1.3	P43277	2	1.11E-02	2.33	Up
Lymphocyte-specific protein 1	P19973	10	2.03E-02	2.15	Up
C-type lectin domain family 4 member F	P70194	12	5.30E-03	2.12	Up
Allograft inflammatory factor 1	O70200	3	5.35E-03	2.11	Up
Alpha-actinin-1	Q7TPR4	20	5.04E-03	2.08	Up
Hematopoietic lineage cell-specific protein	P49710	10	1.66E-02	2.02	Up
EF-hand domain-containing protein D1	Q9D4J1	3	2.92E-02	2.02	Up
Tyrosine-protein phosphatase non-receptor type substrate 1	P97797	2	4.56E-02	1.97	Up
Histone H1.0	P10922	2	1.22E-02	1.87	Up
Annexin A1	P10107	14	1.22E-02	1.86	Up
Integrin alpha-L	P24063	4	1.58E-02	1.74	Up
Prelamin-A/C	P48678	9	2.11E-02	1.52	Up
ATP synthase subunit alpha, mitochondrial	Q03265	16	3.66E-02	1.49	Up
ATP synthase subunit beta, mitochondrial	P56480	25	4.22E-02	1.43	Up
Lysosome-associated membrane glycoprotein 1	P11438	3	4.39E-02	−1.45	Down
Filamin-A	Q8BTM8	59	2.53E-02	−1.57	Down
V-type proton ATPase subunit B, brain isoform	P62814	3	2.16E-02	−1.79	Down
Plectin	Q9QXS1	4	1.65E-02	−1.80	Down
Proliferation-associated protein 2G4	P50580	4	1.91E-02	−1.85	Down
DNA-binding protein A	Q9JKB3	2	3.33E-02	−1.88	Down
Glutathione S-transferase Mu 1	P10649	8	2.57E-02	−1.90	Down
Tubulin alpha-4A chain	P68368	3	1.92E-02	−1.98	Down
Polyadenylate-binding protein 1	P29341	5	6.88E-03	−2.01	Down
Isocitrate dehydrogenase [NADP] cytoplasmic	O88844	4	4.32E-02	−2.18	Down
Lysozyme C-1	P17897	2	8.32E-03	−2.27	Down
Coagulation factor XIII A chain	Q8BH61	13	6.73E-03	−2.99	Down

(n = 3 in each experiment). Protein were considered significant with a Mass Protein Profiler calculated ANOVA p < 0.05 when comparing Ly6C^hi^ and Ly6C^low^.

**Table 4:** DAVID functional pathway enrichment of significantly altered proteins between Ly6C^hi^ and Ly6C^low^ macrophages.

Biological Processes Low vs High					
GO ID	Term	Count	%	PValue	Fold Enrichment
**GO:0019886**	**Antigen Processing and Presentation of Exogenous Peptide Antigen via MHC Class II**	**4**	**8.51**	**0.00001**	**112.31**
**Protein ID**	**Protein Name**	**Peptide #**	**p(Low vs High)**	**Fold Change**	**Regulation**
P14434	H-2 class II histocompatibility antigen, A-B alpha chain	4	0.00046	17.26	Up
P14483	H-2 class II histocompatibility antigen, A beta chain	4	0.00019	15.85	Up
P04441	H-2 class II histocompatibility antigen gamma chain	4	0.00071	4.74	Up
P08101	Low affinity immunoglobulin gamma Fc region receptor II	4	0.01726	2.46	Up
**GO ID**	**Term**	**Count**	**%**	**PValue**	**Fold Enrichment**
**GO:0019882**	**Antigen Processing and Presentation**	**3**	**6.38**	**0.00798**	**21.84**
**Protein ID**	**Protein Name**	**Peptide #**	**p(Low vs High)**	**Fold Change**	**Regulation**
P14434	H-2 class II histocompatibility antigen, A-B alpha chain	4	0.00046	17.26	Up
P14483	H-2 class II histocompatibility antigen, A beta chain	4	0.00019	15.85	Up
P04441	H-2 class II histocompatibility antigen gamma chain	4	0.00071	4.74	Up
**GO ID**	**Term**	**Count**	**%**	**PValue**	**Fold Enrichment**
**GO:0030041**	**Actin Filament Polymerization**	**3**	**6.38**	**0.00161**	**49.14**
**Protein ID**	**Protein Name**	**Peptide #**	**p(Low vs High)**	**Fold Change**	**Regulation**
P13020	Gelsolin	11	0.00136	3.17	Up
O70200	Allograft inflammatory factor 1	3	0.00535	2.11	Up
P49710	Hematopoietic lineage cell-specific protein	10	0.01658	2.02	Up
**GO ID**	**Term**	**Count**	**%**	**PValue**	**Fold Enrichment**
**GO:0031532**	**Actin Cytoskeleton Reorganization**	**3**	**6.38**	**0.00742**	**22.68**
**Protein ID**	**Protein Name**	**Peptide #**	**p(Low vs High)**	**Fold Change**	**Regulation**
P31725	Protein S100-A9	8	0.00004	32.22	Up
P10107	Annexin A1	14	0.01223	1.86	Up
Q8BTM8	Filamin-A	59	0.02534	−1.57	Down
**GO ID**	**Term**	**Count**	**%**	**PValue**	**Fold Enrichment**
**GO:0006911**	**Phagocytosis, Engulfment**	**4**	**8.51**	**0.00019**	**34.94**
**Protein ID**	**Protein Name**	**Peptide #**	**p(Low vs High)**	**Fold Change**	**Regulation**
P13020	Gelsolin	11	0.00136	3.17	Up
P08101	Low affinity immunoglobulin gamma Fc region receptor II	4	0.01726	2.46	Up
O70200	Allograft inflammatory factor 1	3	0.00535	2.11	Up
P97797	Tyrosine-protein phosphatase non-receptor type substrate 1	2	0.04559	1.97	Up
**GO ID**	**Term**	**Count**	**%**	**PValue**	**Fold Enrichment**
**GO:0002376**	**Immune System Process**	**8**	**17.02**	**0.00004**	**8.21**
**Protein ID**	**Protein Name**	**Peptide #**	**p(Low vs High)**	**Fold Change**	**Regulation**
P31725	Protein S100-A9	8	0.00004	32.22	Up
P27005	Protein S100-A8	5	0.00022	31.14	Up
P14434	H-2 class II histocompatibility antigen, A-B alpha chain	4	0.00046	17.26	Up
P14483	H-2 class II histocompatibility antigen, A beta chain	4	0.00019	15.85	Up
P08071	Lactotransferrin	14	0.00046	14.67	Up
P11672	Neutrophil gelatinase-associated lipocalin	2	0.00242	14.47	Up
P04441	H-2 class II histocompatibility antigen gamma chain	4	0.00071	4.74	Up
P10107	Annexin A1	14	0.01223	1.86	Up
**GO ID**	**Term**	**Count**	**%**	**PValue**	**Fold Enrichment**
**GO:0006955**	**Immune Response**	**4**	**8.51**	**0.03003**	**5.78**
**Protein ID**	**Protein Name**	**Peptide #**	**p(Low vs High)**	**Fold Change**	**Regulation**
P14434	H-2 class II histocompatibility antigen, A-B alpha chain	4	0.00046	17.26	Up
P14483	H-2 class II histocompatibility antigen, A beta chain	4	0.00019	15.85	Up
P04441	H-2 class II histocompatibility antigen gamma chain	4	0.00071	4.74	Up
P08101	Low affinity immunoglobulin gamma Fc region receptor II	4	0.01726	2.46	Up
**GO ID**	**Term**	**Count**	**%**	**PValue**	**Fold Enrichment**
**GO:0006954**	**Inflammatory Response**	**5**	**10.64**	**0.01038**	**5.71**
**Protein ID**	**Protein Name**	**Peptide #**	**p(Low vs High)**	**Fold Change**	**Regulation**
P31725	Protein S100-A9	8	0.00004	32.22	Up
P27005	Protein S100-A8	5	0.00022	31.14	Up
O35744	Chitinase-3-like protein 3	13	0.00629	2.54	Up
O70200	Allograft inflammatory factor 1	3	0.00535	2.11	Up
P10107	Annexin A1	14	0.01223	1.86	Up
**GO ID**	**Term**	**Count**	**%**	**PValue**	**Fold Enrichment**
**GO:0045087**	**Innate Immune Response**	**5**	**10.64**	**0.01722**	**4.91**
**Protein ID**	**Protein Name**	**Peptide #**	**p(Low vs High)**	**Fold Change**	**Regulation**
P31725	Protein S100-A9	8	0.00004	32.22	Up
P27005	Protein S100-A8	5	0.00022	31.14	Up
P51437	Cathelin-related antimicrobial peptide	2	0.00002	28.13	Up
P11672	Neutrophil gelatinase-associated lipocalin	2	0.00242	14.47	Up
P10107	Annexin A1	14	0.01223	1.86	Up
**GO ID**	**Term**	**Count**	**%**	**PValue**	**Fold Enrichment**
**GO:0043066**	**Negative Regulation of Apoptotic process**	**6**	**12.77**	**0.01286**	**4.17**
**Protein ID**	**Protein Name**	**Peptide #**	**p(Low vs High)**	**Fold Change**	**Regulation**
P08071	Lactotransferrin	14	0.00046	14.67	Up
P04441	H-2 class II histocompatibility antigen gamma chain	4	0.00071	4.74	Up
O70200	Allograft inflammatory factor 1	3	0.00535	2.11	Up
Q8BTM8	Filamin-A	59	0.02534	−1.57	Down
P50580	Proliferation-associated protein 2G4	4	0.01913	−1.85	Down
Q9JKB3	DNA-binding protein A	2	0.03331	−1.88	Down
**GO ID**	**Term**	**Count**	**%**	**PValue**	**Fold Enrichment**
**GO:0006915**	**Apoptotic Process**	**5**	**10.64**	**0.05269**	**3.45**
**Protein ID**	**Protein Name**	**Peptide #**	**p(Low vs High)**	**Fold Change**	**Regulation**
P31725	Protein S100-A9	8	0.00004	32.22	Up
P27005	Protein S100-A8	5	0.00022	31.14	Up
P11672	Neutrophil gelatinase-associated lipocalin	2	0.00242	14.47	Up
P13020	Gelsolin	11	0.00136	3.17	Up
P19973	Lymphocyte-specific protein 1	10	0.02033	2.15	Up
**Molecular Functions Low vs High**					
**GO ID**	**Term**	**Count**	**%**	**PValue**	**Fold Enrichment**
**GO:0003779**	**Actin Binding**	**8**	**17.02**	**0.00002**	**9.18**
**Protein ID**	**Protein Name**	**Peptide #**	**p(Low vs High)**	**Fold Change**	**Regulation**
P13020	Gelsolin	11	0.00136	3.17	Up
P70460	Vasodilator-stimulated phosphoprotein	2	0.01674	2.91	Up
P19973	Lymphocyte-specific protein 1	10	0.02033	2.15	Up
O70200	Allograft inflammatory factor 1	3	0.00535	2.11	Up
Q7TPR4	Alpha-actinin-1	20	0.00504	2.08	Up
P49710	Hematopoietic lineage cell-specific protein	10	0.01658	2.02	Up
Q8BTM8	Filamin-A	59	0.02534	−1.57	Down
Q9QXS1	Plectin	4	0.01647	−1.80	Down
**GO ID**	**Term**	**Count**	**%**	**PValue**	**Fold Enrichment**
**GO:0051015**	**Actin Filament Binding**	**3**	**6.38**	**0.04371**	**8.81**
**Protein ID**	**Protein Name**	**Peptide #**	**p(Low vs High)**	**Fold Change**	**Regulation**
O70200	Allograft inflammatory factor 1	3	0.00535	2.11	Up
Q7TPR4	Alpha-actinin-1	20	0.00504	2.08	Up
Q8BTM8	Filamin-A	59	0.02534	−1.57	Down
**GO ID**	**Term**	**Count**	**%**	**PValue**	**Fold Enrichment**
**GO:0005509**	**Calcium Ion Binding**	**9**	**19.15**	**0.00031**	**4.99**
**Protein ID**	**Protein Name**	**Peptide #**	**p(Low vs High)**	**Fold Change**	**Regulation**
P31725	Protein S100-A9	8	0.00004	32.22	Up
P27005	Protein S100-A8	5	0.00022	31.14	Up
P13020	Gelsolin	11	0.00136	3.17	Up
Q9D8Y0	EF-hand domain-containing protein D2	3	0.02416	2.79	Up
O70200	Allograft inflammatory factor 1	3	0.00535	2.11	Up
Q7TPR4	Alpha-actinin-1	20	0.00504	2.08	Up
Q9D4J1	EF-hand domain-containing protein D1	3	0.02919	2.02	Up
P10107	Annexin A1	14	0.01223	1.86	Up
P56480	ATP synthase subunit beta, mitochondrial	25	0.04219	1.43	Up

(n = 3 in each experiment). Pathway enrichment was considered significant with a DAVID calculated t-test p < 0.05 when comparing Ly6C^hi^ and Ly6C^low^.

## Discussion

Alcoholic liver disease is a major public health issue and accounts for approximately 48% of liver cirrhosis related deaths^1^. As infiltrating macrophages are known to mediate the pathogenesis of ALD from steatosis to cirrhosis^8–10^, analysis of the altered signaling pathways between the different subsets of these cells in response to ethanol is of the utmost importance in developing treatment options to prevent the progression of ALD or promote the reversal of scar tissue formation in the liver. Macrophages display a remarkable capacity to adapt their phenotype based on tissue micro-environmental cues such as lipid exposure, hypoxia, cytokines, and efferocytosis of apoptotic cells^21,25^. To date, no studies have been conducted providing analysis of the cellular metabolome and proteome of infiltrating liver macrophages isolated from an *in vivo* model of ALD. While several studies have utilized immortalized mouse macrophages (RAW264.7) for transcriptomic^26^ and lipidomic^26–28^ analysis following inflammatory stimuli, this study is the first to look at *in vivo* polarized macrophages in an ALD model, therefore allowing for the natural effects of the tissue microenvironment, such as the gut-liver signaling axis, and ethanol metabolism on regulating liver infiltrating macrophage phenotypes.

It has previously been shown that following phagocytosis of apoptotic hepatocytes, Ly6C^hi^ macrophages differentiate into Ly6C^low^ macrophages which express higher levels of phagocytosis related genes after alcohol intake^21^. In healthy or control diet fed mouse livers, infiltrating macrophages are limited until liver insult elicits the recruitment of Ly6C^+^ monocytes into the liver tissue.^11,21,25^ Therefore, the analysis done in this study was focused on the difference between Ly6C^hi^ and Ly6C^low^ macrophages from ethanol fed mice without comparison to control diet fed animals. In our present study, we observed a significant increase in phagocytosis and engulfment related proteins (Table 4). We detected an upregulation of phagocytosis related proteins in Ly6C^low^ macrophages; this is expected as phagocytosis of apoptotic cells induces an anti-inflammatory phenotype^29,30^. Additionally, we saw a significant enrichment in proteins involved in regulating the apoptotic process. Furthermore, we observed a significant enrichment in actin polymerization and cytoskeletal reorganization in Ly6C^low^ macrophages. Alterations in actin contractility, cytoskeletal organization and cellular elongation have been shown to induce macrophages to an anti-inflammatory phenotype as evidenced by increased arginase-1 and YM-1 expression, hallmarks of anti-inflammatory macrophage polarization^31^. Additionally, defects in actin polymerization have been shown to attenuate macrophage phagocytic ability^32^. This suggests further *in vivo* analysis of actin polymerization and cytoskeletal organization in murine macrophages may elucidate a novel therapeutic strategy in modulating macrophage phenotypes in ALD by affecting macrophage phagocytosis and response to apoptotic stimuli.

Recently Zhang *et al*. provided a comprehensive analysis profiling lipid species during *in vitro* differentiation of mouse and human macrophages cell lines. They reported a significant increase in the composition of glycerophospholipid species during macrophage differentiation. Furthermore, they saw a significant increase in the levels of lysophospholipids in anti-inflammatory macrophages compared to pro-inflammatory macrophages suggesting that modulation of glycerophospholipid metabolism could be a vital signaling component in differentiation of liver macrophage phenotypes^33^. In our study, we found a significant enrichment in glycerophospholpid metabolism with differential metabolite regulation between Ly6C^hi^ and Ly6C^low^ macrophages. Additionally, we observed enrichment for arachidonic acid metabolism and phospholipid biosynthesis (Table 2). In each of the enriched pathways, we detected a massive upregulation in multiple phosphatidylcholine (PC) species in Ly6C^low^ macrophages. PCs has been shown to promote an anti-inflammatory phenotype in macrophages through modulating actin assembly and increasing mycobacterium growth in RAW264.7 and J774 macrophages^34^. Likewise, we observed a substantial upregulation in phosphatidylethanolamine (PE(18:2/18:2) in Ly6C^low^ macrophages. Following stimulation with nonsteroidal anti-inflammatory agents, macrophages have been shown to display an increase in multiple PE species and take on an anti-inflammatory phenotype^35^. Therefore, the observed changes we see in PC and PE species correlate with *in vitro* studies highlighting the anti-inflammatory properties of PC and PE glycerophospholipid species in modulating macrophage phenotypes. Also of interest in regard to PE(18:2/18:2) is the linonleic acid (18:2) constituents present at the *sn-1* and *sn-2* positions, as linoleic acid has been shown to promote an anti-inflammatory phenotype in macrophages^36^. These results suggest the involvement of phospholipase A_2_ (PLA_2_) in regulating macrophage polarization in ALD. PLA_2_ is involved in the hydrolysis of *sn*-2 fatty acids from membrane glycerophospholipids yielding a free fatty acid, arachidonic acid, and a lysophospholipid^37^. The functions of PLA_2_ in modulating the inflammatory response have been well established in a variety of inflammatory contexts^38–42^. Ishihara *et al*. have shown that targeting cytosolic PLA_2_ activity in non-alcoholic fatty liver disease models proved beneficial in preventing hepatic fibrosis formation and reducing hepatocyte death^43,44^. Rodrigues *et al*. showed that using diethylcarbmazine, which modulates arachidonic acid metabolism and cyclooxygenase-2 (COX-2) mediated prostaglandin production, elicited an anti-inflammatory and protective response in ALD^45^. In addition to COX-2 mediated arachidonic acid metabolism and prostaglandin synthesis, arachidonic acid can be metabolized via cytochrome P450 epoxygenase mediated pathway to generate epoxyeicosatrienoic acids (EETs)^46^. We found a significant increase in EETs in the Ly6C^low^ phenotype. Endogenous EETs have been shown to regulate the ability of *in vitro* THP-1 monocytes to differentiate into pro-inflammatory macrophages in response to pro-inflammatory stimuli (lipopolysaccharide (LPS) and interferon γ (IFNγ) as well as preventing differentiation under anti-inflammatory stimuli (IL-4)^46^. Additionally, it has been shown that the immunomodulatory effect of EETs on inducing pro-inflammatory macrophage differentiation was facilitated through attenuation of NF-κB signaling^47^. Finally, studies have shown that eicosatrienoic acid inhibits LPS induced inflammatory gene expression in macrophages^48^. We detected an upregulation of eicosatrienoic acid metabolites in the anti-inflammatory, Ly6C^low^ macrophages after alcohol consumption. These studies coupled with the observed increase in arachidonic acid, glycerophospolipid metabolism and phospholipid biosynthesis as well as increased calcium ion binding suggest future investigation of the role of calcium dependent and independent PLA_2_ activity for therapeutic targeting of macrophage polarization in ALD.

The present study provides a framework for future studies utilizing multi-omics approaches for analyzing signaling difference between pro- and anti-inflammatory macrophages isolated from ALD mouse models. We detected a number of metabolic and protein mediated pathways that were significantly altered between the two macrophage subtypes, validating a number of *in vitro* studies analyzing the lipid, metabolite, and protein profile of polarized macrophages^26–28,33,48^. While the present study utilized an ALD model in which the degree of inflammation is not as evident histopathologically as more aggressive models, such as the NIAAA model, it allowed for the sufficient isolation of infiltrating liver macrophages not normally present in the healthy liver. We identified a number of metabolic pathways significantly altered due to the early onset of alcohol-induced hepatic inflammation (arachidonic acid metabolism, glycerophospholipid metabolism and phospholipid biosynthesis), which suggests that PLA_2_ enzymes play a critical role in modulating macrophage phenotypes. To explore the impact of PLA_2_ on ALD, future studies could utilize whole body PLA_2_ knockout mice or known PLA_2_ pharmacological inhibitors to elucidate the impact of PLA_2_ on macrophage polarization in ALD models. Overall, the data presented here justifies a further need to investigate numerous signaling mechanisms implicated in the modulation of macrophage phenotypes during ALD.

## Materials and Methods

### Animal Model

Female C57BL/6 J mice (The Jackson Laboratory, Bar Harbor, ME, USA) (n = 30) were maintained under pathogen-free conditions in the Center for Laboratory Animal Care at the University of Colorado Anschutz Medical Campus (Aurora, CO, USA). All experiments were performed using an Institutional Animal Care and Use Committee (IACUC) approved protocol and in accordance to the guidelines of the IACUC at the University of Colorado Anschutz Medical Campus. To elicit infiltrating macrophage recruitment to the liver, mice were fed an ethanol-containing Lieber-Decarli liquid diet (Bio-Serv, Flemington, NJ, USA). Ethanol content was introduced gradually by increasing 1.6% (v/v) every 2 days until 5%. All mice were then fed the liquid diet containing 5% ethanol for 4 weeks, as described previously^49,50^.

### Isolation of Liver Non-Parenchymal Cells (NPCs)

Liver NPCs were isolated following a previously described method^51^. Briefly, a 20-G catheter was put through the mouse superior vena cava, the inferior vena cava was clamped, and the portal vein cut. The liver was perfused with Hank’s balanced salt solution (HBSS), followed by a digestion buffer [1 × HBSS, supplemented with 0.04% collagenase (type IV; Sigma, St. Louis, MO, USA), 1.25 mM CaCl_2_, 4 mM MgSO_4_, and 10 mM HEPES]. After digestion, the liver was disrupted in ACD solution (1 × HBSS, supplemented with 0.5% FBS, 0.6% citrate-dextrose solution, and 10 mM HEPES). Single cells were passed through a 100-μm cell strainer, and the cells were fractionated using 30% (w/v) Nycodenz (Axis-Shield PoC AS, Oslo, Norway) at 1.155 g/mL to yield liver NPCs and further purified using 30% Percoll (Sigma) at 1.04 g/mL.

### Flow Cytometry Assisted Cell Sorting (FACS)

To purify KCs, Ly6C^hi^ and Ly6C^low^ IMs, liver NPCs were incubated with normal rat serum (Sigma) and anti-mouse FcγRII/III (Becton Dickinson, Franklin Lakes, NJ, USA) to minimize nonspecific antibody binding. Subsequently, the cells were stained with anti-CD45, anti-Ly6C, anti-Ly6G, anti-CD19, anti-SiglecF (Becton Dickinson) and anti-F4/80, anti-CD11b, anti-NK1.1 and anti-CD3 (eBioscience, San Diego, CA, USA), and sorted using a BD FACSAria II Cell Sorter (BD Bioscience, San Jose, CA, USA).

### Metabolomics Sample Preparation and Analysis

Cell pellets from different sort dates were combined in order to get 3 technical replicates of approximately 400–500 K cells per sample type (Ly6C^hi^ and Ly6C^low^). Extractions were performed using volumes of 70% MeOH/water and 100% MeOH based on cell numbers. Cold methanol was used to precipitate proteins prior to liquid-liquid extraction of metabolites. Proteins pellets were saved for future proteomics analysis. Liquid-liquid extraction was performed on the supernatant using water and methyl tert-butyl ether (MTBE).The aqueous and lipid fractions were retained for analysis. Lipid fractions were analyzed using SB-C18 HPLC analytical column in positive and negative ionization mode on the Agilent 6560 IM-QTOF (in QTOF mode only). Aqueous fractions were analyzed using a HILIC column in positive ionization mode on the Agilent 6560 IM-QTOF (in QTOF mode only). A pooled sample was used as instrument QCs to monitor the entire instrument analysis. Initial data QC, peak threshold evaluation, retention time variation, and charge carrier evaluation was performed in Agilent MassHunter Qualitative Analysis, version B.07.00. Data extraction was performed in MassHunter Profinder, version B.08.00. Differential Analysis was performed in Agilent Mass Profiler Professional (MPP), version 14.5. Compound annotation (database searches and molecular formula generation) was performed in MassHunter ID Browser software, version 14.5. Raw MS data were checked for quality and reproducibility. Appropriate spectral and chromatogram peak height thresholds were determined by careful examination of the raw data. Appropriate charge carriers to be allowed during data extraction were determined after preliminary extraction on selected samples. The Agilent “recursive workflow” was used to prepare data. This workflow includes the following steps: 1) untargeted extraction using the Find-by-Molecular Feature algorithm, 2) mass and time alignment of extracted compounds, 3) targeted extraction using the Find-by-Ion algorithm (using the list of ions prepared in step 1), 4) final mass and time alignment of extracted compounds.

### Metaboanalyst and Metabolites Biological Role (MBROLE) Analysis

For Metaboanalyst comparison the following analysis parameters were used: Mass Tolerance: 0.05, No Missing Value Imputation, Data Filtering: Mean Intensity Value, Sample Normalization: Normalization by Sum, Data Transformation: None, Data Scaling: Mean Centering, Fold Change Threshold: 2, T-test: Group Variance Equal. For MBROLE metabolite functional enrichment analysis, pathways were considered significant with a p < 0.05.

### Proteomics Sample Preparation, nHPLC-MS and nHPLC-MS/MS Analysis

Following methanol extraction for metabolomics, the remaining cell pellets from each technical replicate were processed using the PreOmics iST 8x Kit (Cat # 00001) following the included protocol. Digested macrophage samples were loaded onto a 2 cm PepMAP 100, nanoviper trapping column and chromatographically resolved on-line using a 0.075 × 250 mm, 2.0 µm Acclaim PepMap RSLC reverse phase nano column (Thermo Scientific) using a 1290 Infinity II LC system equipped with a nanoadapter (Agilent). Mobile phases consisted of water + 0.1% formic acid (A) and 90% aq. acetonitrile + 0.1% formic acid (B). Samples were loaded onto the trapping column at 3.2 μL/min for 2.5 minutes at initial condition before being chromatographically separated at an effective flow rate of 345 nl/min using a gradient of 3–8.5% B over 4.0 minutes, 8.5–26% B over 48.5 minutes, and 26–35% over 7.5 minutes for a total 60 minute gradient. The gradient method was followed by a column wash at 70% B for 5 minutes. For nHPLC-MS, data was collected with a 6550 QTOF equipped with a nano source (Agilent) operated in MS mode. For nHPLC-MS/MS, data was collected with a 6550 QTOF equipped with a nano source (Agilent) operated using Data Dependent Acquisition CID Auto MS/MS. The capillary voltage, drying gas flow, and drying gas temperature were set to 1300 V, 11.0 l/min, and 200 C, respectively. Data was collected in positive ion polarity over mass ranges 290–1700 m/z at a scan rate of 1.5 spectra/s. MS/MS scans were collected over mass ranges 50–1700 m/z at a scan rate of 3 spectra/s. Singly charged species were excluded from being selected during MS/MS acquisition. Following data acquisition in MS/MS mode, sample data was searched in SpectrumMill to identify proteins.

### DAVID Bioinformatics Analysis

Functional pathway enrichment of significantly altered proteins between Ly6C^hi^ and Ly6C^low^ macrophage population was analyzed using the Database for Annotation, Visualization and Integrated Discovery (DAVID) Bioinformatics Resource 6.8. For pathway enrichment, significantly altered proteins were compared to the whole mouse background. Pathways were considered significant with at least 3 proteins involved, a fold enrichment >2, and a p < 0.05.

### Statistical Analysis

Statistical analysis of significantly altered metabolites and proteins was determined using Mass Profiler Professional Software. For Metaboanalyst, significantly altered metabolites were determined based of the difference in peak height intensity between the analyze metabolites with a p < 0.05. For MBROLE analysis for metabolite functional pathway enrichment, pathways were considered significant with a p < 0.05. For DAVID pathway enrichment, significantly altered proteins were compared to the whole mouse background. Pathways were considered significant with at least 3 proteins involved, a fold enrichment >2, and a p < 0.05.

## Supplementary information

Supplementary Data
