# Supplementary material for: Multi-omics Analysis of Liver Infiltrating Macrophages Following Ethanol Consumption
Source: Sci Rep. 2019 May 23;9:7776. doi: 10.1038/s41598-019-43240-4 (PMC6533323; doi:10.1038/s41598-019-43240-4)
Supplement: Supplementary file 1 — Supplementary Data [file 41598_2019_43240_MOESM1_ESM.pdf]

Multi-omics Analysis of Liver Infiltrating Macrophages Following Ethanol Consumption

John O. Marentette[1#], Meng Wang[1#], Cole R. Michel[1], Roger Powell[1], Xing Zhang[1], Nichole Reisdorph[1], Kristofer S. Fritz[1] and Cynthia Ju[1]*

[1]Skaggs School of Pharmacy and Pharmaceutical Sciences, University of Colorado Anschutz Medical Campus

Aurora, CO 80045

[#]Authors contributed equally to this study

Corresponding Authors:

Cynthia Ju, Department of Anesthesiology, McGovern Medical School, University of Texas Health Sciences Center at Houston, 6431 Fannin Street, MSB 6.246, Houston, Texas, 77030 (Current Address). Send correspondence to Changqing.Ju@uth.tmc.edu

Kristofer Fritz, Department of Pharmaceutical Sciences, Skaggs School of Pharmacy and Pharmaceutical Sciences, University of Colorado Anschutz Medical Campus, 12850 E. Montview Blvd, C238, Aurora, Colorado, 80045. Send correspondence to Kristofer.Fritz@ucdenver.edu

Supplementary Table 1. Complete List of Differentially Regulated Metabolomics Analysis with Metabolites

Supplementary Table 2. Complete List of Differentially Regulated Proteomic Analysis with Proteins

Supplementary Table 3. UniProt Accession Numbers for Common and Unique Proteins in Ly6C^{hi} and Ly6C^{low} Macrophages.

Supplementary Table 4. Common and Unique Biological Processes for Ly6C^{hi} and Ly6C^{low} Macrophages obtained from submitting UniProt accession number to DAVID.

Supplementary Table 5. Common and Unique Molecular Functions for Ly6C^{hi} and Ly6C^{low} Macrophages obtained from submitting UniProt accession number to DAVID.

Supplementary Table S1

MBROLE Pathway Enrichment Analysis						
**KEGG Pathway**	**Glycerophospholipid metabolism**	**p=0.00000015**				
**Metabolite ID**	**Compound**	**p ([LOW] vs [HI])**	**Regulation**	**FC**	**Mass**	**Retention Time**
HMDB07879	PC(14:0/20:1)	0.00000117	Up	14552.299	759.577	2.7959998
C05973	LysoPE(0:0/20:4)	0.00000004	Up	8191.464	501.2852	1.5950001
C05980	CL(20:4/20:4/18:1/18:1)	0.02237148	Up	2.3857725	1501.0281	5.2099996
C05980	CL(16:0/18:1/18:1/18:0)	0.02935672	Up	2.216448	1433.0435	5.2099996
C02737	PS(22:2/20:4)	0.00619864	Up	2.1309059	863.5636	6.431
C00416	PA(14:0/13:0)	0.01894119	Up	2.03645	614.3692	5.2680006
C02737	PS(18:0/20:3)	0.01018467	Up	1.8234403	813.5564	5.286
C05980	CL(18:0/18:1/18:1/18:0)	0.03703265	Down	-1.5966635	1461.0708	6.4690013
C05980	CL(20:1/18:2/18:1/18:1)	0.02421535	Down	-1.668816	1525.0492	5.209
C05973	LysoPE(0:0/22:5)	0.04909987	Down	-1.7876333	509.2879	1.6539999
C05980	CL(18:0/18:0/18:2/18:0)	0.03732978	Down	-2.0619185	1457.063	5.2099996
HMDB09093	PE(18:2/18:2)	0.00000009	Down	-6742.0083	739.5146	2.8740003
**KEGG Pathway**	**Metabolic pathways**	**p=0.00411**				
**Metabolite ID**	**Compound**	**p ([LOW] vs [HI])**	**Regulation**	**FC**	**Mass**	**Retention Time**
HMDB00374	17-Hydroxyprogesterone	1.92E-09	Up	52550.8	330.2258	1.1479999
HMDB07879	PC(14:0/20:1)	0.00000117	Up	14552.299	759.577	2.7959998
C01885	MG(0:0/18:1/0:0)	4.13E-07	Up	13307.73	378.2748	2.5389998
C05980	CL(20:4/20:4/18:1/18:1)	0.02237148	Up	2.3857725	1501.0281	5.2099996
C05980	CL(16:0/18:1/18:1/18:0)	0.02935672	Up	2.216448	1433.0435	5.2099996
C02737	PS(22:2/20:4)	0.00619864	Up	2.1309059	863.5636	6.431

C00416	PA(14:0/13:0)	0.01894119	Up	2.03645	614.3692	5.2680006
C02737	PS(18:0/20:3)	0.01018467	Up	1.8234403	813.5564	5.286
HMDB04693	11H-14,15-EETA	0.007826052	Up	1.6741626	358.2093	1.2530001
HMDB04264	14,15-Epoxy-5,8,11-eicosatrienoic acid	0.03620125	Up	1.6090974	320.2328	1.1569998
HMDB00305	Vitamin A	0.03911776	Up	1.6040646	286.2295	2.271
HMDB03871	13-L-Hydroperoxylinoleic acid	0.038854554	Up	1.579102	312.2277	1.8080001
C05775	alpha-Ribazole	0.04385764	Up	1.4749023	314.104	1.2270001
C00399	Ubiquinone-4	0.018471733	Up	1.2788694	490.2843	2.4319997
HMDB03464	4-Guanidinobutanoic acid	0.006786303	Down	-1.455486	145.085	4.139
C05980	CL(18:0/18:1/18:1/18:0)	0.03703265	Down	-1.5966635	1461.0708	6.4690013
C05980	CL(20:1/18:2/18:1/18:1)	0.02421535	Down	-1.668816	1525.0492	5.209
HMDB04951	Ceramide (d18:1/20:0)	0.021412965	Down	-2.0462277	593.5757	7.063001
C05980	CL(18:0/18:0/18:2/18:0)	0.03732978	Down	-2.0619185	1457.063	5.2099996
C01885	MG(0:0/18:4/0:0)	0.010899141	Down	-3.0027268	350.2418	1.455
C01885	MG(0:0/18:4/0:0)	0.015869742	Down	-3.9003434	350.2434	1.373
HMDB09093	PE(18:2/18:2)	0.00000009	Down	-6742.0083	739.5146	2.8740003
**KEGG Pathway**	**Arachidonic acid metabolism**	**p=0.00893**				
**Metabolite ID**	**Compound**	**p ([LOW] vs [HI])**	**Regulation**	**FC**	**Mass**	**Retention Time**
HMDB07879	PC(14:0/20:1)	0.00000117	Up	14552.299	759.577	2.7959998
HMDB04693	11H-14,15-EETA	0.007826052	Up	1.6741626	358.2093	1.2530001
HMDB04264	14,15-Epoxy-5,8,11-eicosatrienoic acid	0.03620125	Up	1.6090974	320.2328	1.1569998
**KEGG Pathway**	**Linoleic acid metabolism**	**p=0.00977**				
**Metabolite ID**	**Compound**	**p ([LOW] vs [HI])**	**Regulation**	**FC**	**Mass**	**Retention Time**
HMDB07879	PC(14:0/20:1)	0.00000117	Up	14552.299	759.577	2.7959998
C01885	MG(0:0/18:1/0:0)	4.13E-07	Up	13307.73	378.2748	2.5389998
C00416	PA(14:0/13:0)	0.01894119	Up	2.03645	614.3692	5.2680006
HMDB03871	13-L-Hydroperoxylinoleic acid	0.038854554	Up	1.579102	312.2277	1.8080001
C01885	MG(0:0/18:4/0:0)	0.010899141	Down	-3.0027268	350.2418	1.455
C01885	MG(0:0/18:4/0:0)	0.015869742	Down	-3.9003434	350.2434	1.373
**HMDB Pathway**	**Arachidonic Acid Metabolism**	**p=0.025**				
**Metabolite ID**	**Compound**	**p ([LOW] vs [HI])**	**Regulation**	**FC**	**Mass**	**Retention Time**
C00157	PC(14:0/20:1)	1.17E-06	Up	14552.299	759.577	2.7959998
C00157	PC(20:3/P-18:1)	9.04E-09	Up	9632.273	793.5885	7.3700004
C00157	PC(20:3/P-18:0)	0.010843969	Up	1.9178302	795.6032	7.6989994
HMDB04693	11H-14,15-EETA	0.007826052	Up	1.6741626	358.2093	1.2530001
HMDB04264	14,15-Epoxy-5,8,11-eicosatrienoic acid	0.03620125	Up	1.6090974	320.2328	1.1569998
C00157	PC(14:1/P-18:0)	0.041162275	Down	-1.3820686	751.5357	5.2099996
C00157	PC(14:1/P-18:0)	0.044275247	Down	-1.7250102	751.5351	5.355
**HMDB Pathway**	**Phospholipid Biosynthesis**	**p=0.0000332**				
**Metabolite ID**	**Compound**	**p ([LOW] vs [HI])**	**Regulation**	**FC**	**Mass**	**Retention Time**
C00157	PC(14:0/20:1)	1.17E-06	Up	14552.299	759.577	2.7959998
C00157	PC(20:3/P-18:1)	9.04E-09	Up	9632.273	793.5885	7.3700004
C00350	PE(18:2/18:2)	8.68E-08	Up	6742.0083	739.5146	2.8740003
C02737	PS(22:2/20:4)	0.00619864	Up	2.1309059	863.5636	6.431
C00350	PE(20:1/20:3)	0.008398175	Up	2.1254194	795.5765	6.4339986
C00350	PE(20:1/20:3)	0.008398175	Up	2.1254194	795.5765	6.4339986
C00416	PA(14:0/13:0)	0.01894119	Up	2.03645	614.3692	5.2680006
C00350	PE(20:2/P-18:1)	0.004769958	Up	1.9665164	753.5574	7.0680003
C00157	PC(20:3/P-18:0)	0.010843969	Up	1.9178302	795.6032	7.6989994
C00350	PE(14:0/22:1)	0.02230334	Up	1.8465439	745.5694	5.279
C02737	PS(18:0/20:3)	0.01018467	Up	1.8234403	813.5564	5.286
C00350	PE(24:0/P-16:0)	0.020011874	Up	1.6373577	805.6088	7.485
C00350	PE(22:2/P-18:1)	0.026633823	Up	1.5159866	781.5885	7.5039997
C00157	PC(14:1/P-18:0)	0.041162275	Down	-1.3820686	751.5357	5.2099996
C00350	PE(14:0/16:0)	0.032299943	Down	-1.520349	663.4833	5.243
C00157	PC(14:1/P-18:0)	0.044275247	Down	-1.7250102	751.5351	5.355
C00350	PE(14:1/20:4)	0.009279267	Down	-1.8418627	709.4657	1.068

Supplementary Table S2

Biological Processes Low vs High					
**GO ID**	**Term**	**Count**	**%**	**PValue**	**Fold Enrichment**
**GO:0019886**	**Antigen Processing and Presentation of Exogenous Peptide Antigen via MHC Class II**	**4**	**8.510638**	**5.14E-06**	**112.31056**
**Protein ID**	**Protein Name**	**Peptide #**	**p(Low vs High)**	**Fold Change**	**Regulation**
P14434	H-2 class II histocompatibility antigen, A-B alpha chain	4	0.00046	17.26	Up
P14483	H-2 class II histocompatibility antigen, A beta chain	4	0.00019	15.85	Up
P04441	H-2 class II histocompatibility antigen gamma chain	4	0.00071	4.74	Up
P08101	Low affinity immunoglobulin gamma Fc region receptor II	4	0.01726	2.46	Up
**GO ID**	**Term**	**Count**	**%**	**PValue**	**Fold Enrichment**
**GO:0019882**	**Antigen Processing and Presentation**	**3**	**6.382979**	**0.0079826**	**21.838164**
**Protein ID**	**Protein Name**	**Peptide #**	**p(Low vs High)**	**Fold Change**	**Regulation**
P14434	H-2 class II histocompatibility antigen, A-B alpha chain	4	0.00046	17.26	Up
P14483	H-2 class II histocompatibility antigen, A beta chain	4	0.00019	15.85	Up
P04441	H-2 class II histocompatibility antigen gamma chain	4	0.00071	4.74	Up
**GO ID**	**Term**	**Count**	**%**	**PValue**	**Fold Enrichment**
**GO:0030041**	**Actin Filament Polymerization**	**3**	**6.382979**	**0.0016142**	**49.13587**
**Protein ID**	**Protein Name**	**Peptide #**	**p(Low vs High)**	**Fold Change**	**Regulation**
P13020	Gelsolin	11	0.00136	3.17	Up
O70200	Allograft inflammatory factor 1	3	0.00535	2.11	Up
P49710	Hematopoietic lineage cell-specific protein	10	0.01658	2.02	Up
**GO ID**	**Term**	**Count**	**%**	**PValue**	**Fold Enrichment**
**GO:0031532**	**Actin Cytoskeleton Reorganization**	**3**	**6.382979**	**0.0074202**	**22.678094**
**Protein ID**	**Protein Name**	**Peptide #**	**p(Low vs High)**	**Fold Change**	**Regulation**
P31725	Protein S100-A9	8	0.00004	32.22	Up
P10107	Annexin A1	14	0.01223	1.86	Up
Q8BTM8	Filamin-A	59	0.02534	-1.57	Down
**GO ID**	**Term**	**Count**	**%**	**PValue**	**Fold Enrichment**
**GO:0006911**	**Phagocytosis, Engulfment**	**4**	**8.510638**	**1.90E-04**	**34.941063**
**Protein ID**	**Protein Name**	**Peptide #**	**p(Low vs High)**	**Fold Change**	**Regulation**
P13020	Gelsolin	11	0.00136	3.17	Up
P08101	Low affinity immunoglobulin gamma Fc region receptor II	4	0.01726	2.46	Up
O70200	Allograft inflammatory factor 1	3	0.00535	2.11	Up
P97797	Tyrosine-protein phosphatase non-receptor type substrate 1	2	0.04559	1.97	Up
**GO ID**	**Term**	**Count**	**%**	**PValue**	**Fold Enrichment**
**GO:0015991**	**ATP Metabolic Process**	**3**	**6.382979**	**0.0044482**	**29.481522**
**Protein ID**	**Protein Name**	**Peptide #**	**p(Low vs High)**	**Fold Change**	**Regulation**
Q03265	ATP synthase subunit alpha, mitochondrial	16	0.03665	1.49	Up
P56480	ATP synthase subunit beta, mitochondrial	25	0.04219	1.43	Up
P62814	V-type proton ATPase subunit B, brain isoform	3	0.02158	-1.79	Down
**GO ID**	**Term**	**Count**	**%**	**PValue**	**Fold Enrichment**
**GO:0015991**	**ATP Hydrolysis Coupled Proton Transport**	**3**	**6.382979**	**0.0030445**	**35.735178**
**Protein ID**	**Protein Name**	**Peptide #**	**p(Low vs High)**	**Fold Change**	**Regulation**
Q03265	ATP synthase subunit alpha, mitochondrial	16	0.03665	1.49	Up
P56480	ATP synthase subunit beta, mitochondrial	25	0.04219	1.43	Up
P62814	V-type proton ATPase subunit B, brain isoform	3	0.02158	-1.79	Down
**GO ID**	**Term**	**Count**	**%**	**PValue**	**Fold Enrichment**
**GO:0015992**	**Proton Transport**	**3**	**6.382979**	**0.0091632**	**20.332084**

Protein ID	Protein Name	Peptide #	p(Low vs High)	Fold Change	Regulation
Q03265	ATP synthase subunit alpha, mitochondrial	16	0.03665	1.49	Up
P56480	ATP synthase subunit beta, mitochondrial	25	0.04219	1.43	Up
P62814	V-type proton ATPase subunit B, brain isoform	3	0.02158	-1.79	Down
**GO ID**	**Term**	**Count**	**%**	**PValue**	**Fold Enrichment**
**GO:0050727**	**Regulation of Inflammatory Response**	**3**	**6.382979**	**0.0100966**	**19.332145**
**Protein ID**	**Protein Name**	**Peptide #**	**p(Low vs High)**	**Fold Change**	**Regulation**
P31725	Protein S100-A9	8	0.00004	32.22	Up
P27005	Protein S100-A8	5	0.00022	31.14	Up
P10107	Annexin A1	14	0.01223	1.86	Up
**GO ID**	**Term**	**Count**	**%**	**PValue**	**Fold Enrichment**
**GO:0042102**	**Positive Regulation of T-cell Proliferation**	**3**	**6.382979**	**0.0110705**	**18.425951**
**Protein ID**	**Protein Name**	**Peptide #**	**p(Low vs High)**	**Fold Change**	**Regulation**
O70200	Allograft inflammatory factor 1	3	0.00535	2.11	Up
P10107	Annexin A1	14	0.01223	1.86	Up
P24063	Integrin alpha-L	4	0.01577	1.74	Up
**GO ID**	**Term**	**Count**	**%**	**PValue**	**Fold Enrichment**
**GO:0006952**	**Defense Response**	**4**	**8.510638**	**0.0030002**	**13.554723**
**Protein ID**	**Protein Name**	**Peptide #**	**p(Low vs High)**	**Fold Change**	**Regulation**
P51437	Cathelin-related antimicrobial peptide	2	0.00002	28.13	Up
P04441	H-2 class II histocompatibility antigen gamma chain	4	0.00071	4.74	Up
P08101	Low affinity immunoglobulin gamma Fc region receptor II	4	0.01726	2.46	Up
P19973	Lymphocyte-specific protein 1	10	0.02033	2.15	Up
**GO ID**	**Term**	**Count**	**%**	**PValue**	**Fold Enrichment**
**GO:0071356**	**Cellular Response to Tumor Necrosis Factor**	**3**	**6.382979**	**0.0306336**	**10.720553**
**Protein ID**	**Protein Name**	**Peptide #**	**p(Low vs High)**	**Fold Change**	**Regulation**
P51437	Cathelin-related antimicrobial peptide	2	0.00002	28.13	Up
P11672	Neutrophil gelatinase-associated lipocalin	2	0.00242	14.47	Up
Q9JKB3	DNA-binding protein A	2	0.03331	-1.88	Down
**GO ID**	**Term**	**Count**	**%**	**PValue**	**Fold Enrichment**
**GO:0006935**	**Chemotaxis**	**3**	**6.382979**	**0.0348364**	**9.9937362**
**Protein ID**	**Protein Name**	**Peptide #**	**p(Low vs High)**	**Fold Change**	**Regulation**
P31725	Protein S100-A9	8	0.00004	32.22	Up
P27005	Protein S100-A8	5	0.00022	31.14	Up
P19973	Lymphocyte-specific protein 1	10	0.02033	2.15	Up
**GO ID**	**Term**	**Count**	**%**	**PValue**	**Fold Enrichment**
**GO:0098609**	**Cell-Cell Adhesion**	**4**	**8.510638**	**0.0115586**	**8.3193007**
**Protein ID**	**Protein Name**	**Peptide #**	**p(Low vs High)**	**Fold Change**	**Regulation**
P70460	Vasodilator-stimulated phosphoprotein	2	0.01674	2.91	Up
Q9D8Y0	EF-hand domain-containing protein D2	3	0.02416	2.79	Up
Q9QXS1	Plectin	4	0.01647	-1.80	Down
O88844	Isocitrate dehydrogenase [NADP] cytoplasmic	4	0.04315	-2.18	Down
**GO ID**	**Term**	**Count**	**%**	**PValue**	**Fold Enrichment**
**GO:0006914**	**Autophagy**	**3**	**6.382979**	**0.0486719**	**8.304654**
**Protein ID**	**Protein Name**	**Peptide #**	**p(Low vs High)**	**Fold Change**	**Regulation**
P31725	Protein S100-A9	8	0.00004	32.22	Up
P27005	Protein S100-A8	5	0.00022	31.14	Up
P11438	Lysosome-associated membrane glycoprotein 1	3	0.04388	-1.45	Down
**GO ID**	**Term**	**Count**	**%**	**PValue**	**Fold Enrichment**
**GO:0002376**	**Immune System Process**	**8**	**17.02128**	**4.11E-05**	**8.2106936**

Protein ID	Protein Name	Peptide #	p(Low vs High)	Fold Change	Regulation
P31725	Protein S100-A9	8	0.00004	32.22	Up
P27005	Protein S100-A8	5	0.00022	31.14	Up
P14434	H-2 class II histocompatibility antigen, A-B alpha chain	4	0.00046	17.26	Up
P14483	H-2 class II histocompatibility antigen, A beta chain	4	0.00019	15.85	Up
P08071	Lactotransferrin	14	0.00046	14.67	Up
P11672	Neutrophil gelatinase-associated lipocalin	2	0.00242	14.47	Up
P04441	H-2 class II histocompatibility antigen gamma chain	4	0.00071	4.74	Up
P10107	Annexin A1	14	0.01223	1.86	Up
**GO ID**	**Term**	**Count**	**%**	**PValue**	**Fold Enrichment**
**GO:0006955**	**Immune Response**	**4**	**8.510638**	**0.0300349**	**5.7806905**
**Protein ID**	**Protein Name**	**Peptide #**	**p(Low vs High)**	**Fold Change**	**Regulation**
P14434	H-2 class II histocompatibility antigen, A-B alpha chain	4	0.00046	17.26	Up
P14483	H-2 class II histocompatibility antigen, A beta chain	4	0.00019	15.85	Up
P04441	H-2 class II histocompatibility antigen gamma chain	4	0.00071	4.74	Up
P08101	Low affinity immunoglobulin gamma Fc region receptor II	4	0.01726	2.46	Up
**GO ID**	**Term**	**Count**	**%**	**PValue**	**Fold Enrichment**
**GO:0006954**	**Inflammatory Response**	**5**	**10.6383**	**0.0103846**	**5.7134732**
**Protein ID**	**Protein Name**	**Peptide #**	**p(Low vs High)**	**Fold Change**	**Regulation**
P31725	Protein S100-A9	8	0.00004	32.22	Up
P27005	Protein S100-A8	5	0.00022	31.14	Up
O35744	Chitinase-3-like protein 3	13	0.00629	2.54	Up
O70200	Allograft inflammatory factor 1	3	0.00535	2.11	Up
P10107	Annexin A1	14	0.01223	1.86	Up
**GO ID**	**Term**	**Count**	**%**	**PValue**	**Fold Enrichment**
**GO:0045087**	**Innate Immune Response**	**5**	**10.6383**	**0.0172154**	**4.913587**
**Protein ID**	**Protein Name**	**Peptide #**	**p(Low vs High)**	**Fold Change**	**Regulation**
P31725	Protein S100-A9	8	0.00004	32.22	Up
P27005	Protein S100-A8	5	0.00022	31.14	Up
P51437	Cathelin-related antimicrobial peptide	2	0.00002	28.13	Up
P11672	Neutrophil gelatinase-associated lipocalin	2	0.00242	14.47	Up
P10107	Annexin A1	14	0.01223	1.86	Up
**GO ID**	**Term**	**Count**	**%**	**PValue**	**Fold Enrichment**
**GO:0043066**	**Negative Regulation of Apoptotic process**	**6**	**12.76596**	**0.0128558**	**4.1669995**
**Protein ID**	**Protein Name**	**Peptide #**	**p(Low vs High)**	**Fold Change**	**Regulation**
P08071	Lactotransferrin	14	0.00046	14.67	Up
P04441	H-2 class II histocompatibility antigen gamma chain	4	0.00071	4.74	Up
O70200	Allograft inflammatory factor 1	3	0.00535	2.11	Up
Q8BTM8	Filamin-A	59	0.02534	-1.57	Down
P50580	Proliferation-associated protein 2G4	4	0.01913	-1.85	Down
Q9JKB3	DNA-binding protein A	2	0.03331	-1.88	Down
**GO ID**	**Term**	**Count**	**%**	**PValue**	**Fold Enrichment**
**GO:0006915**	**Apoptotic Process**	**5**	**10.6383**	**0.0526859**	**3.4481312**
**Protein ID**	**Protein Name**	**Peptide #**	**p(Low vs High)**	**Fold Change**	**Regulation**
P31725	Protein S100-A9	8	0.00004	32.22	Up
P27005	Protein S100-A8	5	0.00022	31.14	Up
P11672	Neutrophil gelatinase-associated lipocalin	2	0.00242	14.47	Up
P13020	Gelsolin	11	0.00136	3.17	Up
P19973	Lymphocyte-specific protein 1	10	0.02033	2.15	Up
**Molecular Functions Low vs High**					
**GO ID**	**Term**	**Count**	**%**	**PValue**	**Fold Enrichment**
**GO:0017124**	**SH3 Domain Binding**	**3**	**6.382979**	**0.0356794**	**9.8564972**
**Protein ID**	**Protein Name**	**Peptide #**	**p(Low vs High)**	**Fold Change**	**Regulation**

P70460	Vasodilator-stimulated phosphoprotein	2	0.01674	2.91	Up
P49710	Hematopoietic lineage cell-specific protein	10	0.01658	2.02	Up
P97797	Tyrosine-protein phosphatase non-receptor type substrate 1	2	0.04559	1.97	Up
**GO ID**	**Term**	**Count**	**%**	**PValue**	**Fold Enrichment**
**GO:0003779**	**Actin Binding**	**8**	**17.02128**	**1.99E-05**	**9.1760684**
**Protein ID**	**Protein Name**	**Peptide #**	**p(Low vs High)**	**Fold Change**	**Regulation**
P13020	Gelsolin	11	0.00136	3.17	Up
P70460	Vasodilator-stimulated phosphoprotein	2	0.01674	2.91	Up
P19973	Lymphocyte-specific protein 1	10	0.02033	2.15	Up
O70200	Allograft inflammatory factor 1	3	0.00535	2.11	Up
Q7TPR4	Alpha-actinin-1	20	0.00504	2.08	Up
P49710	Hematopoietic lineage cell-specific protein	10	0.01658	2.02	Up
Q8BTM8	Filamin-A	59	0.02534	-1.57	Down
Q9QXS1	Plectin	4	0.01647	-1.80	Down
**GO ID**	**Term**	**Count**	**%**	**PValue**	**Fold Enrichment**
**GO:0051015**	**Actin Filament Binding**	**3**	**6.382979**	**0.0437143**	**8.8111111**
**Protein ID**	**Protein Name**	**Peptide #**	**p(Low vs High)**	**Fold Change**	**Regulation**
O70200	Allograft inflammatory factor 1	3	0.00535	2.11	Up
Q7TPR4	Alpha-actinin-1	20	0.00504	2.08	Up
Q8BTM8	Filamin-A	59	0.02534	-1.57	Down
**GO ID**	**Term**	**Count**	**%**	**PValue**	**Fold Enrichment**
**GO:0098641**	**Cadherin Binding Involved in Cell-Cell Adhesion**	**6**	**12.76596**	**6.58E-04**	**8.3373955**
**Protein ID**	**Protein Name**	**Peptide #**	**p(Low vs High)**	**Fold Change**	**Regulation**
P70460	Vasodilator-stimulated phosphoprotein	2	0.01674	2.91	Up
Q9D8Y0	EF-hand domain-containing protein D2	3	0.02416	2.79	Up
P10107	Annexin A1	14	0.01223	1.86	Up
Q8BTM8	Filamin-A	59	0.02534	-1.57	Down
Q9QXS1	Plectin	4	0.01647	-1.80	Down
O88844	Isocitrate dehydrogenase [NADP] cytoplasmic	4	0.04315	-2.18	Down
**GO ID**	**Term**	**Count**	**%**	**PValue**	**Fold Enrichment**
**GO:0032403**	**Protein Complex Binding**	**5**	**10.6383**	**0.0124088**	**5.4146493**
**Protein ID**	**Protein Name**	**Peptide #**	**p(Low vs High)**	**Fold Change**	**Regulation**
P06797	Cathepsin L1	4	2.77E-08	5875.09	Up
Q99JI6	Ras-related protein Rap-1b	2	0.04547	1512.80	Up
P49710	Hematopoietic lineage cell-specific protein	10	0.01658	2.02	Up
P24063	Integrin alpha-L	4	0.01577	1.74	Up
Q8BTM8	Filamin-A	59	0.02534	-1.57	Down
**GO ID**	**Term**	**Count**	**%**	**PValue**	**Fold Enrichment**
**GO:0005509**	**Calcium Ion Binding**	**9**	**19.14894**	**3.15E-04**	**4.9917024**
**Protein ID**	**Protein Name**	**Peptide #**	**p(Low vs High)**	**Fold Change**	**Regulation**
P31725	Protein S100-A9	8	0.00004	32.22	Up
P27005	Protein S100-A8	5	0.00022	31.14	Up
P13020	Gelsolin	11	0.00136	3.17	Up
Q9D8Y0	EF-hand domain-containing protein D2	3	0.02416	2.79	Up
O70200	Allograft inflammatory factor 1	3	0.00535	2.11	Up
Q7TPR4	Alpha-actinin-1	20	0.00504	2.08	Up
Q9D4J1	EF-hand domain-containing protein D1	3	0.02919	2.02	Up
P10107	Annexin A1	14	0.01223	1.86	Up
P56480	ATP synthase subunit beta, mitochondrial	25	0.04219	1.43	Up
**GO ID**	**Term**	**Count**	**%**	**PValue**	**Fold Enrichment**
**GO:0044822**	**Poly(A) RNA Binding**	**9**	**19.14894**	**0.0060683**	**3.1349506**
**Protein ID**	**Protein Name**	**Peptide #**	**p(Low vs High)**	**Fold Change**	**Regulation**
P40630	Transcription factor A, mitochondrial	2	0.00901	3.09	Up
P43277	Histone H1.3	2	0.01107	2.33	Up

P10922	Histone H1.0	2	0.01215	1.87	Up
Q03265	ATP synthase subunit alpha, mitochondrial	16	0.03665	1.49	Up
Q8BTM8	Filamin-A	59	0.02534	-1.57	Down
Q9QXS1	Plectin	4	0.01647	-1.80	Down
P50580	Proliferation-associated protein 2G4	4	0.01913	-1.85	Down
Q9JKB3	DNA-binding protein A	2	0.03331	-1.88	Down
P29341	Polyadenylate-binding protein 1	5	0.00688	-2.01	Down
**GO ID**	**Term**	**Count**	**% Coverage**	**P-value**	**Fold Enrichment**
**GO:0042803**	**Protein Homodimerization Activity**	**6**	**12.76596**	**0.0493247**	**2.9149541**
**Protein ID**	**Protein Name**	**Peptide #**	**p(Low vs High)**	**Fold Change**	**Regulation**
P11672	Neutrophil gelatinase-associated lipocalin	2	0.00242	14.47	Up
Q7TPR4	Alpha-actinin-1	20	0.00504	2.08	Up
P10107	Annexin A1	14	0.01223	1.86	Up
Q8BTM8	Filamin-A	59	0.02534	-1.57	Down
P10649	Glutathione S-transferase Mu 1	8	0.02571	-1.90	Down
O88844	Isocitrate dehydrogenase [NADP] cytoplasmic	4	0.04315	-2.18	Down
**GO ID**	**GO Term**	**Count**	**% Coverage**	**P-value**	**Fold Enrichment**
**GO:0005515**	**Protein Binding**	**26**	**55.31915**	**1.96E-06**	**2.4633214**
**Protein ID**	**Protein Name**	**Peptide #**	**p(Low vs High)**	**Fold Change**	**Regulation**
P06797	Cathepsin L1	4	2.77E-08	5875.09	Up
Q99JI6	Ras-related protein Rap-1b	2	0.04547	1512.80	Up
P14434	H-2 class II histocompatibility antigen, A-B alpha chain	4	0.00046	17.26	Up
P14483	H-2 class II histocompatibility antigen, A beta chain	4	0.00019	15.85	Up
P11672	Neutrophil gelatinase-associated lipocalin	2	0.00242	14.47	Up
P04441	H-2 class II histocompatibility antigen gamma chain	4	0.00071	4.74	Up
P13020	Gelsolin	11	0.00136	3.17	Up
P70460	Vasodilator-stimulated phosphoprotein	2	0.01674	2.91	Up
Q9D8Y0	EF-hand domain-containing protein D2	3	0.02416	2.79	Up
O09044	Synaptosomal-associated protein 23	2	0.00440	2.51	Up
P08101	Low affinity immunoglobulin gamma Fc region receptor II	4	0.01726	2.46	Up
P43277	Histone H1.3	2	0.01107	2.33	Up
O70200	Allograft inflammatory factor 1	3	0.00535	2.11	Up
Q7TPR4	Alpha-actinin-1	20	0.00504	2.08	Up
P49710	Hematopoietic lineage cell-specific protein	10	0.01658	2.02	Up
P97797	Tyrosine-protein phosphatase non-receptor type substrate 1	2	0.04559	1.97	Up
P10107	Annexin A1	14	0.01223	1.86	Up
P48678	Prelamin-A/C	9	0.02113	1.52	Up
Q03265	ATP synthase subunit alpha, mitochondrial	16	0.03665	1.49	Up
P56480	ATP synthase subunit beta, mitochondrial	25	0.04219	1.43	Up
P11438	Lysosome-associated membrane glycoprotein 1	3	0.04388	-1.45	Down
Q8BTM8	Filamin-A	59	0.02534	-1.57	Down
Q9QXS1	Plectin	4	0.01647	-1.80	Down
Q9JKB3	DNA-binding protein A	2	0.03331	-1.88	Down
P10649	Glutathione S-transferase Mu 1	8	0.02571	-1.90	Down
P29341	Polyadenylate-binding protein 1	5	0.00688	-2.01	Down

Supplementary Table S3

**Proteins**		
**Common**	**Low**	**High**
**UniProt Accesion #**	**UniProt Accesion #**	**UniProt Accesion #**
Q5DTY9	Q9QZ05	Q8K1I7
Q6GQT1	Q9D5S7	Q8K4B0
P49722	Q9WV54	Q6NVF9
Q6ZQ06	Q5SUA5	Q9D2R6
Q8BJ05	P01635	Q3UPI1

O70133	Q61647	Q6VGS5
P63158	Q8BUH8	Q6PB66
Q9EPB4	Q61096	P01872
P62748	P51437	Q9DB15
P05202	Q3V016	P51885
Q9CRB9	P11531	Q6PB70
Q9ESX5	Q8BZB2	P35486
Q9CPP6	Q9JJV4	P63085
Q6WVG3	Q6P5E4	Q9DBZ5
P63276	Q5XG71	P35374
P17751	Q9CZU3	Q9ET54
Q9DCS9	Q3UZ39	P70315
P43277	Q80VP2	O08648
Q4KML4	Q9CRB2	Q9R1E6
Q9CQN1	Q60764	Q9CWJ9
Q8BLY1	P97426	Q9Z0X4
P70349	P02831	P27048
Q9DBT9	P41245	Q61753
Q3U9G9	Q9JIS5	Q99JT2
P14115	P32067	Q9D7G4
P68254	P13541	Q8VHQ4
Q9CQ62	Q99LX5	O88623
Q9WV32	Q91V79	Q9CYR0
Q9Z130	Q8R2S8	Q9D868
P70248	P16381	Q4JK59
Q921T2	Q8BYK6	Q9DB05
P01887	Q9D023	P50636
Q922Q8	Q3V1U8	Q80TY5
P14869	O35144	B1AXH1
P63168	Q99PF4	Q9CPV4
P58771	P61027	Q8BND5
P31725	Q8K371	Q5BL07
Q62393	Q8BMQ3	Q6PDQ2
Q60710	Q9QUN9	O54750
Q9Z2I8	P56812	Q8R0G7
P14824	Q80YR5	A1L314
Q3UND0	Q9R0U0	Q6IFX2
Q9CXW4	P70460	Q8CGC4
P68369	Q61288	Q3TLH4
P97370	Q8C255	O35286
Q99KC8	Q6VYH9	P62874
P24527	P11672	Q8CBW3
O09111	P17679	P62814
P07724	P29699	Q9QYJ0
P01899	Q3UHK3	Q9QZD9
Q62523	Q9JLN9	P10711
Q9CPT0	Q9CZ44	Q80XP9

Q60864	Q8BHH9	Q8BH70
P01898	P97823	Q91V92
Q9QUH0	Q62440	P56399
Q99KJ8	P55288	Q99N48
P47911	P28352	Q6KAU4
Q9JLJ2	P10078	P70423
Q6ZWU9	Q8BR26	P62830
P29391	O88668	Q9Z1T1
Q9EQU5	P97494	P97369
P52480	Q3V0A6	Q60692
Q9D1G1	Q61644	P25976
P11438	Q63850	A2AQ25
P56480	P14483	P14685
Q00623	Q64524	P62754
P02535	P26011	Q03958
P56395	P14873	Q9D2V7
Q9EST5	P97298	Q8VDG3
Q921I1	Q9QZD5	Q9QXS1
Q9DBY8	P20917	P55302
Q9ERL7	Q7TQI3	O35691
Q9CVB6	Q9CSU0	Q91YI6
P46638	Q9DC69	O09167
Q8BK64	P97797	Q80UP5
P80313	O55098	Q9WVE8
P97821	Q9R0Q3	P70419
P51859	Q5SSH7	Q9EQQ3
P51410	Q8K4K6	Q8BQ47
P80318	A2RT91	Q9CYL5
P02088	Q9CPY7	P43024
Q9JJ94	Q61792	Q5PRF0
Q3U7R1	P70333	P27512
P84075	Q9DCT2	Q3USB7
Q60605	Q80YN3	P62717
Q8CJG1	Q91V41	P01592
P42932	Q61838	Q8K0B3
Q9ERS2	P08071	P07146
P62852	Q9QXN0	Q9CPX9
Q9JM76	Q32M07	Q91VM9
Q99K48	Q8JZP2	P27641
P06467	P14901	Q9QXA5
P40142	Q9JLT4	P62862
Q8VD58	Q9D7V9	P30416
P08905	O08808	Q63870
P01027	Q00PI9	P54728
P12815	Q61136	Q8CCS6
Q3TTY5	Q9JI57	Q9D6Z1
Q7TPW1	Q5QNV8	Q3THK7

P00405	Q01147	O70252
Q9CR21	P49300	Q91WJ8
P09671	Q8BPB0	Q8BWQ4
Q61171	Q9CRB5	Q05921
Q61316	Q3UXZ6	P49813
Q60932	Q11136	Q4U4S6
P16110	Q80WE4	Q9CWW6
Q921H8	Q8K389	Q9R0P3
Q8BTM8	Q7TN31	P31649
P16546	Q9DAS9	P62313
P20491	Q8K0B2	P97801
Q8CGP5	Q9DBU3	Q6P7W0
P25444	Q8VE22	Q9DBR1
Q641P0	Q8VCW8	Q5SX40
Q4VAA2	Q8BLB7	Q5SUV2
P97372	Q8BIK4	Q9R1N9
P61961	Q04447	Q9D0W5
P62774	Q9CRC8	Q61074
P38647	Q8BJS4	Q8JZK9
P43275	P61957	Q9QZS0
P06151	P97452	A2BDX3
P51881	Q99KP6	Q9D898
P06800	Q61646	P26516
Q9R0Q7	O70200	Q9ERN0
Q922R8	P28078	Q3UVD5
Q91WV0	B1AXP6	P59325
Q8K561	P45376	P01837
P62908	P21460	Q9D1R9
P19536	Q7TQA3	Q5HZK1
O08553	Q6A026	Q3UFY8
Q99MR6	Q9WVB4	P62918
Q9DBA6	P07309	Q00417
Q8R0W0	Q811D2	P56593
P97351	Q8BUI3	Q8R1B4
P21803	Q8BUM9	Q99KN9
O88342	Q3UP87	Q9CYD3
P51174	Q9JJL3	Q3V079
P56959	P10922	P62960
Q9CQ75	O55222	Q99NB9
Q3THW5	Q60902	Q01768
P62264	Q60973	P46735
P57780	Q04859	Q9Z1P6
P17918	Q9CW03	Q920A5
Q3UV17	Q9DAI4	Q9Z1Y9
P84228	Q8CDK2	O88643
Q9D8E6	Q9EPN1	Q68ED7
Q9CQA3	Q9CQ19	P29416

P20060	Q9D5V5	P06684
P62821	Q3U4G3	P09066
P20029	Q3UW53	Q9ERA0
P31996	O88593	Q8BGV7
Q9CQQ7	Q9DBY0	Q8BLQ0
P62281	Q8BFR5	P59470
O70492	Q62189	Q8BIL5
P27546	O55125	Q9JLV6
P63101	P43430	Q9D2L1
Q9Z0H4	Q8R344	Q8C437
Q8QZT1	Q9Z0E6	P62334
P52503	P49290	Q9CQ01
Q5PR68	Q922X9	Q9CR61
P62204	Q9Z180	Q62028
P60867	P63005	P16460
P29788	P69525	Q922B2
Q8K310	O35280	Q3UQN2
P50518	Q8K4I3	P81269
Q6ZWY3	Q80X80	A2AJ76
P68433	Q8BUK6	Q8BP47
Q9DBL2	Q8BJM3	Q62504
Q9D8N0	Q9D8C4	P31230
P70372	O08539	Q2VWQ2
Q99JY9	Q9CQI7	Q9JJI8
P84078	Q148W0	Q5RKZ7
Q8C196	A2AAE1	P27040
P63017	O70340	P70232
A2AGT5	Q9DCG9	Q02257
Q9JKB3	P43345	O35943
Q9Z2U0	P63254	Q3TZX8
P59999	Q07417	Q91ZZ5
P07356	Q7TSC3	Q9ERI2
O08573	Q8BL97	Q9CQZ6
P38060	P68134	Q99388
Q91VW3	Q920B9	Q9D8U8
Q8BP67	P61226	P52196
Q9CPW4	Q9D416	P70677
Q9QUK6	P15247	P21995
P27600	Q9CZE3	A6H6Q4
Q99KF1	P29758	Q62376
Q61696	Q6NZJ6	B1AZP2
P54071	Q60949	Q63886
A2ASS6	Q8VEM8	A2APY7
P01029	P18761	Q9JMG1
Q9EQ20	Q8BK63	Q8R2G6
Q3TGF2	Q8C5L3	Q9DCM0
Q6ZWM4	P26323	Q9R190

P62806	Q6ZWR6	Q8BXJ8
P46660	Q02395	P70195
Q61425	P04441	Q9CXW3
P58774	Q148V8	P14404
P12970	Q6A065	Q91VS7
Q8CHT0	P11369	Q9WTU3
P11859	P62855	Q8R0V5
P67984	O89112	Q9JHI5
P11499	Q8CG71	Q91XU3
P99024	Q9CRB6	Q9CR95
P00493	Q9CY25	O08912
Q62425	Q8VCA8	Q91WD5
Q9CQU0	Q9DAR7	Q9DCH4
Q6PHN9	O54824	P28033
O89086	Q9DCN2	Q99LI2
P62858	Q80W04	Q9JL62
Q61462	P60469	P35294
Q80ZW0	Q5HZG4	P15092
P62492	Q3V0I2	Q9CQL1
O35744	Q9QXK3	O70400
Q9Z1Q5	Q8CD19	Q8C9X1
P47963		Q8BSQ9
P08752		Q9CQC7
Q9D0E1		P62245
P12787		Q9CQB5
P68372		Q2TV84
Q9R1P4		Q8C186
Q8VEK3		A3KG59
Q6NZB0		P33267
O70591		Q05A36
O88653		Q8K2H4
O35226		P36536
O08583		P11416
Q61703		P22892
Q99MD9		Q3UPL0
Q9D1D4		P49962
Q64152		Q91VR8
Q62318		P58389
P53026		O35367
O70493		Q99LB7
P15116		O55143
O09131		Q60996
P50396		Q01730
P40630		O35709
Q8BFR4		Q8C3J5
Q9CZM2		Q8JZN7
Q05920		Q9CQK7

Q9D2L6		P46656
P01863		Q91X72
P19973		Q99LY2
Q8BK67		P50136
Q3UBX0		Q99JX7
Q61781		Q8BHE5
P08003		O08784
P30681		Q9CQE8
P60710		P50446
Q9DB77		Q62148
Q9WTP7		Q4U2R1
P15379		Q9D0V7
Q9D6J5		P01631
P80317		Q8BJ63
P09528		Q921U8
Q9ERD7		O08663
Q60931		Q9QXD6
Q91YM2		Q8CGM2
Q8BV49		Q6PCQ0
P11247		Q9CPR5
P68040		Q9Z1D1
P50516		Q68FF7
P84104		P86048
Q9QZ23		P70158
P14131		Q9CWK8
P50580		Q80TI0
P24270		P17665
O70404		Q62446
P18181		O35295
P99027		Q71FD7
Q8VCT4		P35329
P57784		Q9QZM2
Q9D6R2		Q9CQM9
P45878		P11276
P16045		Q9D0B6
P14148		Q9R1P3
Q9CQR2		P31254
P06745		Q61686
P28656		Q3V0B4
P10605		Q80V31
Q9D4J1		Q6ZPY7
Q3V0K9		Q99M15
P62082		P61082
O70370		Q91VW5
Q9R159		P70444
P70441		Q9QYS9
Q99020		Q66JS6

Q9CY58		Q6ZWQ0
P58252		Q9Z2K1
P19157		P25233
Q921Z5		Q8C015
Q3UHX2		Q8K4Z3
P62751		Q9JKV1
Q9Z204		Q80UX8
Q05144		Q9DBH5
Q61735		P52760
P62984		Q8CHY6
Q9CZY3		O55128
Q61207		Q8R326
P14434		Q9EQ32
P83917		Q9ERG0
P05555		P60330
P08101		Q3UL97
Q8K1Z0		P80314
O70145		P48725
O35459		P39749
P67778		P45700
P47753		Q9Z2T6
Q61545		P99028
Q8BGD9		O08807
Q8CII2		Q8C0S4
O35841		Q60787
Q921M7		Q80V03
Q61166		Q9D051
Q8VE97		Q08879
P62242		Q9JKP5
Q61599		Q9CQW9
P70696		P51175
Q9D0J8		Q9QWY8
Q8CIN4		Q0V8T8
P14211		Q6R3M4
Q9D3D9		Q9QXT0
Q9CR16		Q99P27
P63001		Q91VS8
Q62093		Q80XA0
P46471		P27870
Q9CQI6		Q9ERH7
P34022		Q65Z40
P61358		Q6ZWX6
Q9D0T1		Q80ZJ1
P61161		Q8R3B1
O35658		P52482
Q9DCD0		Q8CA95
P09411		P62320

Q9WTP6		Q9Z0P5
Q99LP6		Q9CQE5
P26443		Q504N7
Q9D8Y0		Q07076
P27005		Q80W93
Q68FD5		Q8C996
O08749		
P28293		
O35892		
P61255		
Q9CZ13		
Q3TEA8		
A2RSQ0		
O08709		
P22599		
P07091		
Q9CQ92		
Q62422		
P03930		
P99029		
Q9R0P5		
Q9CR68		
Q9CQ60		
P28076		
Q99LC5		
O08585		
Q8CFW1		
P80315		
P70670		
Q78IK2		
P28063		
P48771		
P97429		
Q6IFZ6		
Q64213		
Q9CPR4		
P62311		
P26041		
Q62383		
P62075		
P11725		
Q8VDW0		
P11352		
Q61937		
Q9JKR6		
P25688		
Q3U0V1		

Q8BHF7		
P15331		
P99026		
P70296		
Q9WVA4		
Q9D8U6		
P81117		
P11983		
P62827		
Q64012		
Q9CQI3		
P09405		
Q80Y14		
P48036		
O08677		
Q60972		
P62737		
P08113		
Q60865		
Q08093		
P14206		
Q9EPK2		
P62962		
P61979		
Q80TR4		
Q99JI4		
Q8R081		
Q99L13		
Q6ZWN5		
Q8BH95		
O70435		
P26645		
Q91YQ5		
Q8BH61		
Q9CQQ8		
Q9ET01		
P17156		
P68368		
Q64467		
Q9CZS1		
P60843		
Q91VA0		
P01864		
Q9DCJ5		
Q8BHZ0		
Q5SWY7		
P21619		

Q9DAU1		
P18242		
Q64727		
Q61879		
Q9JHJ0		
P97807		
P07901		
Q9D0M3		
Q9CQV8		
P03995		
Q9CQ71		
Q60847		
Q61081		
Q7TPR4		
Q99L47		
Q9WV55		
Q9DBG6		
Q8CFI0		
P62835		
P97371		
P59108		
Q8BG07		
Q501J6		
O35114		
P14152		
Q9Z1N5		
P48962		
Q99PL5		
Q9CXI5		
P04104		
Q61033		
P17897		
Q9ESD7		
P35278		
Q62241		
Q64522		
O08638		
P97352		
P49710		
Q64433		
P13020		
Q9CXZ1		
P49935		
Q00612		
Q7TMK9		
P34884		
P63330		

P01942		
Q9QWR8		
Q60749		
Q6PNC0		
P43274		
Q9D0B0		
P57759		
P17742		
P24063		
Q62186		
P11835		
Q62192		
Q9DBJ1		
Q9WV80		
O35479		
P68510		
P53810		
Q8VDD5		
P63028		
P47757		
P62259		
O55022		
Q61990		
Q9WUU7		
P20152		
P54227		
P56391		
P97855		
P97822		
Q99KI0		
P35980		
P09055		
Q06185		
Q9D1J3		
P62137		
Q922U2		
O89053		
Q9D8B3		
O88685		
O70251		
Q8CI43		
Q99JI6		
P60766		
Q9CPX8		
P51863		
Q8C1B7		
P32020		

Q9CYZ2		
P31001		
P29351		
Q8CIH5		
P62900		
Q03265		
Q8VH51		
P24668		
Q9QUI0		
P62301		
P08226		
P42225		
Q9D6P8		
Q62418		
P16858		
P40124		
Q9WUM4		
Q9WUK2		
P09103		
Q9JL26		
Q99PN3		
P63242		
P17225		
Q6ZWY6		
P43276		
Q9JKF1		
Q9JLC8		
P50543		
Q91VI7		
Q8K386		
Q8BMK4		
Q91W90		
O35639		
P35564		
P47738		
P63038		
P15532		
P10107		
P51150		
P10126		
P26369		
Q6P5H2		
Q9ESP1		
Q6P069		
P29341		
Q8K3J1		
Q8QZY9		

Q9CQX2		
P67871		
Q9CQ22		
P21107		
O88569		
Q99KV1		
Q9CXE7		
Q8K4Z5		
Q02053		
Q5XJY5		
Q9R111		
P50544		
P45952		
P84096		
Q7TMM9		
Q9D6J6		
P62309		
O35737		
Q62009		
Q01853		
P47955		
O54879		
P28740		
P60335		
Q9Z2U1		
Q9JJW6		
P24369		
P17047		
P63213		
Q60930		
P19253		
P26040		
P15864		
Q61820		
Q9DB20		
P31786		
P05213		
Q8VCI0		
P61089		
Q8CHH9		
P97493		
Q61093		
Q80X41		
Q7TQK5		
Q91XA2		
Q9QZQ8		
O35900		

P97427		
P61963		
Q8BFZ3		
Q00519		
P84099		
Q99PT1		
P42125		
Q6PDM2		
O08795		
P61982		
O70439		
Q9CZP5		
P11031		
P05064		
Q60648		
Q9R1C8		
Q8CCK0		
Q9D154		
Q9WUM3		
P18760		
P14733		
Q8BWT1		
Q9Z2X1		
P54869		
P08207		
Q9D2G2		
Q3THE2		
Q9QY76		
Q8R1V4		
P49312		
Q9D1A2		
P19783		
P20108		
Q9Z0M6		
P16675		
O88531		
Q8VBT6		
O88844		
Q61390		
Q9D855		
Q8VED5		
Q61656		
Q9D0M5		
O09159		
O35685		
Q9JIW9		
O08997		

Q9CZU6		
Q9CY50		
Q9CXU9		
P62307		
Q64475		
Q8VIJ6		
Q9CQS8		
P97461		
P97384		
O35381		
Q8CH18		
Q99L45		
Q05816		
P26039		
Q91Z31		
P47791		
Q99LX0		
Q7TNV0		
P68037		
P60060		
P47915		
P23198		
Q9QUM9		
Q99JY0		
Q3URU2		
Q9CWZ3		
P97315		
Q9DCM7		
P63280		
P08249		
P62270		
Q61233		
Q61029		
Q921F2		
Q6IRU2		
P17182		
P70168		
P35979		
Q8BG05		
O09061		
Q01339		
P57776		
P60882		
P63325		
P12710		
P27773		
Q8VE70		

Q61704		
Q62426		
Q8CEC0		
Q9DCX2		
Q9CZX8		
Q60668		
P63323		
P10649		
Q60631		
Q9CWH6		
O88456		
P47754		
P28867		
P62897		
Q93092		
Q8K4L4		
P62702		
P61939		
O70445		
Q9CR51		
O54962		
P47962		
Q6PGC1		
Q91WN1		
Q9R1P0		
Q9CPQ1		
Q9DCW4		
O55135		
Q9JJU8		
Q78ZA7		
Q7TMQ7		
Q80TY0		
Q8BMS1		
P24452		
Q9D0S9		
Q9DBP5		
P27659		
Q61598		
P32261		
P35700		

Supplementary Table S4

**Biological Processes**
**Common**
oxidation-reduction process
mast cell activation
platelet aggregation
positive regulation of binding

response to endoplasmic reticulum stress
apical junction assembly
negative regulation of substrate adhesion-dependent cell spreading
positive regulation of substrate adhesion-dependent cell spreading
negative regulation of endothelial cell proliferation
positive regulation of ATPase activity
establishment of protein localization to membrane
removal of superoxide radicals
ribosomal small subunit assembly
maturation of SSU-rRNA
response to L-ascorbic acid
response to zinc ion
positive regulation of catalytic activity
2-oxoglutarate metabolic process
mRNA export from nucleus
ER to Golgi vesicle-mediated transport
cellular response to thyroid hormone stimulus
ribosomal subunit export from nucleus
defense response to Gram-negative bacterium
actin filament polymerization
oxaloacetate metabolic process
positive regulation of telomerase RNA localization to Cajal body
superoxide metabolic process
negative regulation of mRNA splicing, via spliceosome
positive regulation of T cell activation
melanosome transport
positive regulation of hydrolase activity
receptor internalization
positive regulation of T cell mediated cytotoxicity
respiratory electron transport chain
regulation of interferon-gamma-mediated signaling pathway
regulation of actin filament polymerization
translational initiation
positive regulation of neuron projection development
regulation of translation
ATP biosynthetic process
nuclear migration
response to oxidative stress
positive regulation of telomere maintenance via telomerase
activated T cell proliferation
positive regulation of NF-kappaB transcription factor activity
liver development
mitochondrial electron transport, ubiquinol to cytochrome c
NADH metabolic process
leukocyte cell-cell adhesion
positive regulation of translation
protein transport
translation
mitochondrial ATP synthesis coupled proton transport
angiotensin mediated vasoconstriction involved in regulation of systemic arterial blood pressure
regulation of cell shape
positive regulation of lamellipodium assembly
response to salt stress
positive regulation of establishment of protein localization to telomere
positive regulation of intrinsic apoptotic signaling pathway
nucleosome assembly
regulation of fibroblast migration
positive regulation of NIK/NF-kappaB signaling
positive regulation of DNA binding
bone resorption
pentose-phosphate shunt
response to activity

hydrogen peroxide biosynthetic process
negative regulation of cell death
small GTPase mediated signal transduction
carbohydrate metabolic process
actin filament capping
mRNA splicing, via spliceosome
gland morphogenesis
ribosomal small subunit export from nucleus
platelet degranulation
erythrocyte development
cell redox homeostasis
histone H3-K27 trimethylation
actin filament organization
negative regulation of reactive oxygen species biosynthetic process
intermediate filament organization
hydrogen peroxide catabolic process
IRES-dependent viral translational initiation
ruffle organization
protein refolding
chaperone-mediated protein folding
response to alkaloid
transport
cholesterol biosynthetic process
platelet formation
protein folding
cellular response to acid chemical
actin filament network formation
receptor-mediated endocytosis
negative regulation of neuron projection development
ribosomal large subunit assembly
positive regulation of type IIa hypersensitivity
negative regulation of phagocytosis
isocitrate metabolic process
binding of sperm to zona pellucida
superoxide anion generation
maturation of LSU-rRNA
ATP hydrolysis coupled proton transport
negative regulation of Arp2/3 complex-mediated actin nucleation
cellular response to organic cyclic compound
ribosomal small subunit biogenesis
actin filament severing
tricarboxylic acid cycle
lipid homeostasis
rRNA processing
mRNA splice site selection
leukocyte migration involved in inflammatory response
mitotic spindle organization
organ regeneration
negative regulation of neuron apoptotic process
positive regulation of protein localization to Cajal body
wound healing, spreading of cells
histone exchange
mRNA transport
liver regeneration
hydrogen peroxide metabolic process
positive regulation of phagocytosis
response to nutrient
maintenance of protein location
endocytosis
negative regulation of protein export from nucleus
positive regulation of gene expression
positive regulation of stress fiber assembly

toxin transport
CRD-mediated mRNA stabilization
cellular response to interleukin-4
response to cold
positive regulation of DNA metabolic process
ribosome biogenesis
protein targeting
positive regulation of superoxide anion generation
regulation of ruffle assembly
response to lipopolysaccharide
cardiac muscle fiber development
positive regulation of actin filament depolymerization
negative regulation of translation
ATP synthesis coupled proton transport
lipid metabolic process
gluconeogenesis
negative regulation of RNA splicing
positive regulation of DNA replication
actin cytoskeleton organization
substantia nigra development
positive regulation of actin filament polymerization
establishment of Golgi localization
cytoskeleton organization
establishment of endothelial barrier
regulation of actin polymerization or depolymerization
translational elongation
regulation of anion transport
cell proliferation
nucleocytoplasmic transport
RNA splicing
alternative mRNA splicing, via spliceosome
response to bacterium
muscle contraction
cytoplasmic translation
negative regulation of apoptotic process
carboxylic acid metabolic process
fatty acid beta-oxidation using acyl-CoA dehydrogenase
phagocytosis, engulfment
immunoglobulin mediated immune response
glycolytic process
activation of cysteine-type endopeptidase activity involved in apoptotic process
fatty acid metabolic process
barbed-end actin filament capping
early endosome to late endosome transport
positive regulation by host of viral process
actin crosslink formation
astral microtubule organization
positive regulation of mRNA splicing, via spliceosome
vasodilation
actin cytoskeleton reorganization
antigen processing and presentation
malate metabolic process
negative regulation of blood coagulation
positive regulation of axon extension
positive regulation of immune response
positive regulation of MAPK cascade
mitochondrial electron transport, cytochrome c to oxygen
positive regulation of protein serine/threonine kinase activity
positive regulation of interleukin-6 secretion
metabolic process
immune system process
ubiquitin-dependent protein catabolic process

negative regulation of cellular component movement
regulation of alternative mRNA splicing, via spliceosome
response to reactive oxygen species
cell-cell adhesion
retina homeostasis
intracellular protein transport
proton transport
positive regulation of extrinsic apoptotic signaling pathway
mitotic spindle assembly
antigen processing and presentation of exogenous peptide antigen via MHC class I, TAP-dependent
protein homotetramerization
regulation of actin cytoskeleton organization
protein stabilization
protein export from nucleus
in utero embryonic development
chaperone-mediated autophagy
ATP metabolic process
transferrin transport
virion assembly
microtubule-based process
aging
response to drug
actin filament bundle assembly
actin filament fragmentation
response to ethanol
nucleosome positioning
pentose-phosphate shunt, oxidative branch
cell-matrix adhesion
response to stress
positive regulation by host of viral genome replication
protein homooligomerization
positive regulation of nitric oxide biosynthetic process
response to cadmium ion
phagocytosis
regulation of inflammatory response
ribosomal large subunit export from nucleus
positive regulation of receptor-mediated endocytosis
proteolysis
integrin-mediated signaling pathway
fatty acid beta-oxidation
response to calcium ion
respiratory burst
negative regulation of inflammatory response to antigenic stimulus
Arp2/3 complex-mediated actin nucleation
mitochondrial electron transport, NADH to ubiquinone
3'-UTR-mediated mRNA stabilization
response to hydrogen peroxide
mRNA processing
proteolysis involved in cellular protein catabolic process
triglyceride metabolic process
positive regulation of cholesterol esterification
positive regulation of adaptive immune response
muscle filament sliding
astrocyte development
glutathione metabolic process
epithelial cell differentiation
osteoblast differentiation
ribosomal large subunit biogenesis
response to heat
**Low**
negative regulation of gene expression
negative regulation of protein phosphorylation

glyoxylate cycle
defense response to fungus
negative regulation of endothelial cell migration
lipid storage
cytosolic transport
positive regulation of cyclin-dependent protein serine/threonine kinase activity involved in G2/M transition of mitotic cell cycle
establishment of protein localization to chromatin
glyceraldehyde-3-phosphate biosynthetic process
negative regulation of histone H3-K27 methylation
regulation of release of sequestered calcium ion into cytosol
protein import into nucleus
maturation of SSU-rRNA from tricistronic rRNA transcript (SSU-rRNA, 5.8S rRNA, LSU-rRNA)
negative regulation of peptidase activity
cellular response to nerve growth factor stimulus
regulation of mRNA splicing, via spliceosome
glyceraldehyde-3-phosphate metabolic process
positive regulation of oxidative phosphorylation uncoupler activity
positive regulation of cysteine-type endopeptidase activity involved in apoptotic process
DNA replication-dependent nucleosome assembly
positive regulation of DNA repair
'de novo' pyrimidine nucleobase biosynthetic process
plasminogen activation
heterotypic cell-cell adhesion
nucleus localization
cellular process
cellular response to fibroblast growth factor stimulus
Rap protein signal transduction
protein targeting to lysosome involved in chaperone-mediated autophagy
regulation of protein kinase activity
positive regulation of angiogenesis
positive regulation of lipid biosynthetic process
uropod organization
cellular response to hydrogen peroxide
response to angiotensin
pentose biosynthetic process
positive regulation of glycoprotein metabolic process
cellular response to glucose stimulus
antigen processing and presentation of exogenous peptide antigen via MHC class II
cell-substrate adhesion
establishment of nucleus localization
ruffle assembly
defense response to Gram-positive bacterium
positive regulation of protein folding
lymphocyte aggregation
negative regulation of growth of symbiont in host
erythrocyte homeostasis
collagen catabolic process
endonucleolytic cleavage to generate mature 3'-end of SSU-rRNA from (SSU-rRNA, 5.8S rRNA, LSU-rRNA)
negative regulation of mature B cell apoptotic process
iron ion homeostasis
regulation of actin filament depolymerization
anion homeostasis
COPII-coated vesicle budding
protein localization to microtubule
cellular response to low-density lipoprotein particle stimulus
positive regulation of gene expression, epigenetic
positive regulation of protein phosphorylation
fatty acid catabolic process
negative regulation of interleukin-23 production
response to selenium ion
response to electrical stimulus
positive regulation of T cell differentiation

positive regulation of I-kappaB kinase/NF-kappaB signaling
negative regulation of protein catabolic process
RNA transport
Golgi organization
neutrophil aggregation
nucleobase-containing compound metabolic process
positive regulation of dendritic spine development
meiotic cytokinesis
response to axon injury
positive regulation of cell migration
cellular response to platelet-derived growth factor stimulus
chaperone mediated protein folding requiring cofactor
cellular protein complex localization
antigen processing and presentation of peptide antigen
antigen processing and presentation of peptide or polysaccharide antigen via MHC class II
blood vessel endothelial cell migration
positive regulation of protein processing in phagocytic vesicle
eukaryotic translation initiation factor 4F complex assembly
intermediate filament cytoskeleton organization
nucleus organization
positive regulation of G1/S transition of mitotic cell cycle
movement of cell or subcellular component
cell migration
cell aging
negative regulation of cell growth
dosage compensation
positive regulation of peptidyl-tyrosine phosphorylation
neuron projection morphogenesis
regulation of androgen receptor signaling pathway
positive regulation of tumor necrosis factor production
regulation of axonogenesis
cellular response to mechanical stimulus
cellular protein localization
skeletal muscle tissue regeneration
positive regulation of dendritic spine morphogenesis
negative regulation of early endosome to late endosome transport
RNA secondary structure unwinding
ribose phosphate biosynthetic process
positive regulation of innate immune response
negative regulation of intrinsic apoptotic signaling pathway in response to DNA damage by p53 class mediator
**High**
vesicle organization
hemoglobin metabolic process
mRNA catabolic process
chaperone-mediated protein complex assembly
acetyl-CoA biosynthetic process from pyruvate
regulation of protein catabolic process
cellular response to organic substance
skeletal muscle myosin thick filament assembly
positive regulation of viral genome replication
negative regulation of cell motility
citrate metabolic process
negative regulation of protein dephosphorylation
aerobic respiration
positive regulation of telomerase activity
actin polymerization or depolymerization
ganglioside catabolic process
positive regulation of establishment of protein localization to plasma membrane
vesicle-mediated transport
brain renin-angiotensin system
negative regulation of microtubule polymerization
regulation of microvillus length

protein homotrimerization
mitochondrial respiratory chain complex I assembly
innate immune response
lipid catabolic process
response to growth hormone
glycosaminoglycan metabolic process
positive regulation of reactive oxygen species biosynthetic process
regulation of translational initiation
spliceosomal snRNP assembly
cytokine secretion
stress fiber assembly
arginine biosynthetic process
histone H3-K4 trimethylation
prostate gland development
complement activation, classical pathway
protein peptidyl-prolyl isomerization
apoptotic process
acetyl-CoA metabolic process
regulation of stress-activated MAPK cascade
lamellipodium morphogenesis
lens fiber cell development
formation of translation preinitiation complex
positive regulation of RNA splicing
apoptotic cell clearance
positive regulation of cell-substrate adhesion
establishment or maintenance of cell polarity
positive regulation of phosphoprotein phosphatase activity
Rac protein signal transduction
single organismal cell-cell adhesion
mitochondrial acetyl-CoA biosynthetic process from pyruvate
neuron projection development
glucose metabolic process
positive regulation of protein targeting to membrane
keratinocyte development
actin filament-based movement
response to organic cyclic compound
midgut development
retinoid metabolic process
rhythmic process
microvillus assembly
positive regulation of podosome assembly
reactive oxygen species metabolic process
protein tetramerization
positive regulation of lamellipodium morphogenesis
electron transport chain
B cell receptor signaling pathway
regulation of apoptotic process
ATP-dependent chromatin remodeling
response to vitamin A
spliceosomal complex assembly
response to toxic substance
cellular response to oxidative stress
scaRNA localization to Cajal body
regulation of G1/S transition of mitotic cell cycle

Supplementary Table S5

**Molecular Functions**
**Common**
**GO TERM**

integrin binding
structural constituent of ribosome
cytochrome-c oxidase activity
oxidoreductase activity, acting on the CH-OH group of donors, NAD or NADP as acceptor
carboxylic acid binding
cell adhesion molecule binding
nitric-oxide synthase regulator activity
ATPase activity
hydrogen ion transmembrane transporter activity
enzyme binding
S100 protein binding
ATP-dependent RNA helicase activity
transferase activity, transferring acyl groups other than amino-acyl groups
calcium-dependent protein binding
MHC class II protein complex binding
supercoiled DNA binding
ubiquinol-cytochrome-c reductase activity
oxygen binding
catalytic activity
protein-disulfide reductase (glutathione) activity
proton-transporting ATPase activity, rotational mechanism
motor activity
mRNA binding
oxidoreductase activity
RNA binding
porin activity
proton-transporting ATP synthase activity, rotational mechanism
calcium-dependent phospholipid binding
core promoter binding
peptidase activator activity involved in apoptotic process
protease binding
nucleobase-containing compound kinase activity
Arp2/3 complex binding
heat shock protein binding
mRNA 3'-UTR binding
ATPase binding
translation elongation factor activity
monosaccharide binding
protein C-terminus binding
protein kinase B binding
ribosomal large subunit binding
nucleosomal DNA binding
protein kinase regulator activity
poly(A) RNA binding
ubiquitin protein ligase binding
unfolded protein binding
acyl-CoA dehydrogenase activity

peroxidase activity
5S rRNA binding
GTPase activity
Rho GDP-dissociation inhibitor binding
pre-mRNA binding
oxidoreductase activity, acting on the aldehyde or oxo group of donors, NAD or NADP as acceptor
thioredoxin peroxidase activity
phosphoprotein binding
cadherin binding involved in cell-cell adhesion
acetyl-CoA C-acyltransferase activity
GDP binding
NADH dehydrogenase activity
NADH dehydrogenase (ubiquinone) activity
protein homodimerization activity
superoxide-generating NADPH oxidase activity
peptide binding
protein binding
ion channel binding
poly(A) binding
ADP binding
oxidoreductase activity, acting on the CH-CH group of donors
glycoprotein binding
lipid binding
kinase binding
kininogen binding
TBP-class protein binding
keratin filament binding
NADP binding
enoyl-CoA hydratase activity
heme binding
large ribosomal subunit rRNA binding
flavin adenine dinucleotide binding
ATPase activity, coupled
rRNA binding
ribonucleoprotein complex binding
protein domain specific binding
carbohydrate binding
endopeptidase activity
protein binding involved in protein folding
beta-N-acetylhexosaminidase activity
histone deacetylase binding
heparin binding
Hsp90 protein binding
NAD binding
GTP binding
MHC class I protein binding
small ribosomal subunit rRNA binding

Rho GTPase binding
nucleotide binding
iron-sulfur cluster binding
actin filament binding
dATP binding
RAGE receptor binding
structural molecule activity
protein complex binding
fatty-acyl-CoA binding
double-stranded RNA binding
voltage-gated anion channel activity
ribosome binding
endopeptidase inhibitor activity
structural constituent of cytoskeleton
ATP binding
chromatin DNA binding
laminin binding
5.8S rRNA binding
peroxiredoxin activity
3-hydroxyacyl-CoA dehydrogenase activity
identical protein binding
isomerase activity
structural constituent of muscle
actin binding
fibroblast growth factor binding
phospholipase A2 inhibitor activity
protein disulfide oxidoreductase activity
2 iron, 2 sulfur cluster binding
antioxidant activity
calcium ion binding
telomerase RNA binding
phospholipase inhibitor activity
protein phosphatase binding
phospholipid binding
ribosomal small subunit binding
protein kinase binding
DNA binding, bending
translation initiation factor activity
cytoskeletal protein binding
oxidoreductase activity, acting on NAD(P)H
protein kinase C binding
chaperone binding
protein disulfide isomerase activity
protein N-terminus binding
aldehyde dehydrogenase (NAD) activity
electron carrier activity
poly(U) RNA binding

single-stranded DNA binding
threonine-type endopeptidase activity
Rac GTPase binding
oxidoreductase activity, acting on the CH-CH group of donors, with a flavin as acceptor
protein heterodimerization activity
mRNA CDS binding
tau protein binding
GTPase binding
**Low**
**GO TERM**
telomerase activity
lipid transporter activity
nitric-oxide synthase binding
lamin binding
SH3 domain binding
low-density lipoprotein particle receptor binding
myosin V binding
myosin heavy chain binding
microtubule binding
peptidase activity
N6-methyladenosine-containing RNA binding
cytokine binding
histone binding
adenylate kinase activity
Hsp70 protein binding
receptor binding
NF-kappaB binding
dynein intermediate chain binding
hydrolase activity, acting on carbon-nitrogen (but not peptide) bonds
lipopolysaccharide binding
oxidoreductase activity, acting on a sulfur group of donors, NAD(P) as acceptor
dipeptidase activity
protein antigen binding
**High**
**GO TERM**
U6 snRNA binding
FK506 binding
drug binding
hydrolase activity
proteasome-activating ATPase activity
fatty acid binding
actin-dependent ATPase activity
phosphoric diester hydrolase activity
endopeptidase activator activity
small GTPase binding
ferric iron binding
GTPase activating protein binding

damaged DNA binding
single-stranded RNA binding
protein self-association
chloride channel inhibitor activity
intermediate filament binding
4 iron, 4 sulfur cluster binding
phospholipase binding
transferrin receptor binding
pyruvate dehydrogenase (NAD+) activity
U1 snRNP binding
U2 snRNA binding
histone methyltransferase binding
phosphatidylinositol-4,5-bisphosphate binding
regulatory region RNA binding
transcription factor binding
peptidyl-prolyl cis-trans isomerase activity
